# TWEAK favors phosphate-induced calcification of vascular smooth muscle cells through canonical and non-canonical activation of NF*κ*B

**DOI:** 10.1038/cddis.2016.220

**Published:** 2016-07-21

**Authors:** L Hénaut, A B Sanz, D Martin-Sanchez, S Carrasco, R Villa-Bellosta, G Aldamiz-Echevarria, Z A Massy, M D Sanchez-Nino, A Ortiz

**Affiliations:** 1 Nephrology, IIS-Fundación Jiménez Díaz, School of Medicine, Universidad Autónoma de Madrid, Madrid 28040, Spain; 2 REDINREN, Madrid 28040, Spain; 3 Cardiac Surgery, IIS-Fundación Jiménez Díaz, School of Medicine, Universidad Autónoma de Madrid, Madrid 28040, Spain; 4 Division of Nephrology, Ambroise Paré University Hospital, APHP, University of Paris Ouest -Versailles-Saint-Quentin-en-Yvelines (UVSQ), Boulogne-Billancourt, Paris, France; 5 INSERM U1018, Research Centre in Epidemiology and Population Health (CESP) Team 5, Villejuif, Paris, France; 6 Fundación Renal Iñigo Alvarez de Toledo-IRSIN, Madrid 28040, Spain

## Abstract

Vascular calcification (VC) is associated with increased cardiovascular mortality in aging, chronic kidney disease (CKD), type 2 diabetes mellitus (T2DM) and atherosclerosis. TNF-like weak inducer of apoptosis (TWEAK) recently emerged as a new biomarker for the diagnosis and prognosis of cardiovascular diseases. TWEAK binding to its functional receptor Fn14 was reported to promote several steps of atherosclerotic plaque progression. However, no information is currently available on the role of TWEAK/Fn14 on the development of medial calcification, which is highly prevalent in aging, CKD and T2DM. This study explored the involvement of TWEAK in human vascular smooth muscle cells (h-VSMCs) calcification *in vitro*. We report that TWEAK binding to Fn14 promotes inorganic phosphate-induced h-VSMCs calcification, favors h-VSMCs osteogenic transition, decreasing acta2 and myh11 and increasing bmp2 mRNA and tissue non-specific alkaline phosphatase (TNAP), and increases MMP9 activity. Blockade of the canonical NF*κ*B pathway reduced by 80% TWEAK pro-calcific properties and decreased osteogenic transition, TNAP and MMP9 activity. Blockade of non-canonical NF*κ*B signaling by a siRNA targeting RelB reduced by 20% TWEAK pro-calcific effects and decreased TWEAK-induced loss of h-VSMCs contractile phenotype and MMP9 activity, without modulating bmp2 mRNA or TNAP activity. Inhibition of ERK1/2 activation by a MAPK kinase inhibitor did not influence TWEAK pro-calcific properties. Our results suggest that TWEAK/Fn14 directly favors inorganic phosphate-induced h-VSMCs calcification by activation of both canonical and non-canonical NF*κ*B pathways. Given the availability of neutralizing anti-TWEAK strategies, our study sheds light on the TWEAK/Fn14 axis as a novel therapeutic target in the prevention of VC.

Vascular calcification (VC) is characterized by the accumulation of calcium and phosphate salts within the cardiovascular system. VC can develop in the intima and media of vessels, as well as in cardiac valves and is associated with an increased cardiovascular mortality.^1^ VC results from an imbalance between calcification inducers (e.g., mineral disorders, inflammation) and inhibitors (e.g., Fetuin A, Matrix Gla Protein, pyrophosphate (PPi)).^2^ Increased inorganic phosphate (Pi) or Ca^2+^ levels, inflammation or vascular injury lead to osteo-chondrogenic conversion of vascular smooth muscle cells (VSMCs), resident fibroblasts or quiescent valve interstitial cells, which newly express runt-related transcription factor 2, tissue non-specific alkaline phosphatase (TNAP) and bone morphogenetic protein 2 (BMP2). This is associated with the release of pro-calcific matrix vesicles and extracellular matrix remodeling (type I collagen synthesis and elastin degradation). In addition, this pro-calcific environment promotes vascular cell apoptosis and the release of apoptotic bodies, which are able to nucleate hydroxyapatite.^3^

VC is highly prevalent in aging, chronic kidney disease (CKD), type 2 diabetes mellitus (T2DM) and atherosclerosis. Aging^4^ and CKD^5^ are associated with low-grade chronic systemic inflammation and inflammation exacerbates age-related diseases, such as atherosclerosis and T2DM.^6^ Recent studies provided compelling evidence that VC is associated with inflammation and is enhanced by certain inflammatory cytokines. However, the precise cytokines and, hence what the therapeutic targets might be, have not been adequately characterized. In particular, some members of the TNF superfamily promote Pi-induced VSMC osteogenic transition and subsequent calcification.^7^ TNF-like weak inducer of apoptosis (TWEAK) is a TNF superfamily cytokine that recently emerged as a new biomarker for diagnosis and prognosis in cardiovascular diseases.^8^ Membrane-bound TWEAK can be processed by furin to soluble TWEAK (sTWEAK).^9^ TWEAK binding to its receptor fibroblast growth factor-inducible molecule 14 (Fn14) activates mitogen-activated protein kinase (MAPK) signaling and promotes NF*κ*B activation by both canonical and non-canonical pathways. As a consequence, TWEAK regulates in a cell-type- and microenvironment-dependent manner cell proliferation, migration, differentiation, death, inflammation, fibrosis and angiogenesis.^10, 11, 12, 13^

TWEAK is expressed by normal and pathological arterial walls.^14^ Although TWEAK can be upregulated after injury,^15^ changes in its gene expression are usually moderate. By contrast, Fn14 expression in the healthy vasculature is usually low or undetectable. However, it is rapidly and highly upregulated under pathological conditions, as described in aortic aneurisms and carotid atherosclerotic plaques, sensitizing to TWEAK effects.^14, 16^ Data from animal models indicate that TWEAK/Fn14 contributes to abdominal aortic aneurysm formation.^17^ Besides, the TWEAK/Fn14 axis promotes several steps of atherosclerotic plaque development, including initiation, progression, destabilization/rupture and subsequent thrombosis.^8^ In particular, in ApoE KO mice, anti-TWEAK treatment decreased inflammation associated with atherosclerotic plaque progression, including the final step of plaque calcification.^18^

Although these data suggest a role for the TWEAK/Fn14 axis in the pathophysiology of intimal calcification in atherosclerosis, it is unclear whether decreased atherosclerotic plaque calcification in anti-TWEAK-treated mice is the result of lower atherosclerotic burden or of a direct effect of TWEAK on intimal calcification. Furthermore, no information is currently available on the role of TWEAK/Fn14 on medial calcification, as observed in CKD and other conditions. The present study aimed to evaluate the potential of TWEAK to directly promote human VSMCs (h-VSMCs) calcification *in vitro.*

## Results

### TWEAK/Fn14 axis favors Pi-induced calcification and h-VSMCs osteogenic transition

**Figure 1:**
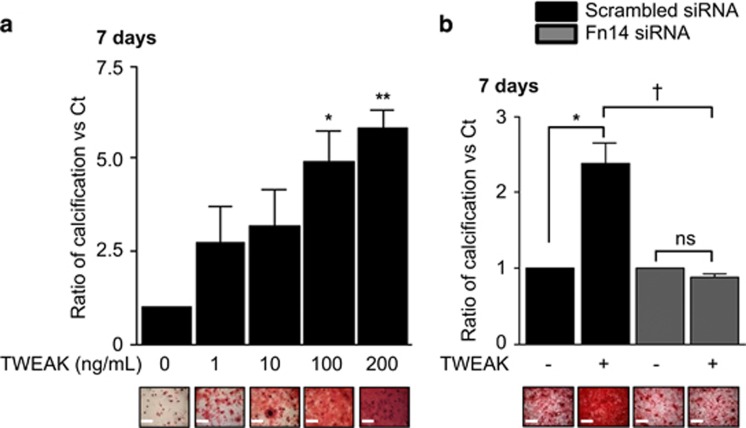
TWEAK/Fn14 promotes Pi-induced h-VSMCs calcification through Fn14 activation. (**a**) Effect of TWEAK on Pi-induced mineral deposition (Alizarin red staining). h-VSMCss were cultured for 7 days in pro-calcific conditions (3.0 mmol/l Pi) in the presence or absence of TWEAK (1–200 ng/ml) in medium containing 1% FBS. Scale bars: 500 *μ*m. *P<0.05 and **P<0.01 *vs* cells exposed to 3.0 mmol/l Pi without TWEAK. Results represent three independent experiments performed in triplicate. Error bars represent the S.E.M. (**b**) Impact of Fn14 downregulation on TWEAK-induced h-VSMCs calcification in pro-calcific conditions (Alizarin red staining). Scale bars: 500 *μ*m. *P<0.05 vs scrambled transfected cells without TWEAK. ^†^*P*<0.05 vs scrambled transfected cells exposed to TWEAK. Results represent three independent experiments performed in triplicate. Error bars represent the S.E.M. NS, not significant

To explore the ability of TWEAK to modulate h-VSMCs mineralization, cells were incubated for 7 days in pro-calcific conditions (3 mmol/l Pi) in combination with increasing concentrations of TWEAK (1–200 ng/ml). In the presence of 3 mmol/l Pi, TWEAK significantly increased mineral deposition in a concentration-dependent manner as assessed by Alizarin red staining (Figure 1a). TWEAK did not show any effect on h-VSMCs calcification when Pi was not added to the culture media (Supplementary Figure S1). Further experiments used 100 ng/ml TWEAK as it was the lowest concentration of TWEAK significantly favoring Pi-induced calcification, although a trend was observed from 1 ng/ml. h-VSMCs viability and proliferation were not affected by Pi or by TWEAK (Supplementary Figure S2). Downregulation of Fn14 by a siRNA (Supplementary Figure S3A) abolished TWEAK effects on Pi-induced calcification as compared with scrambled transfected cells (Figure 1b).

**Figure 2:**
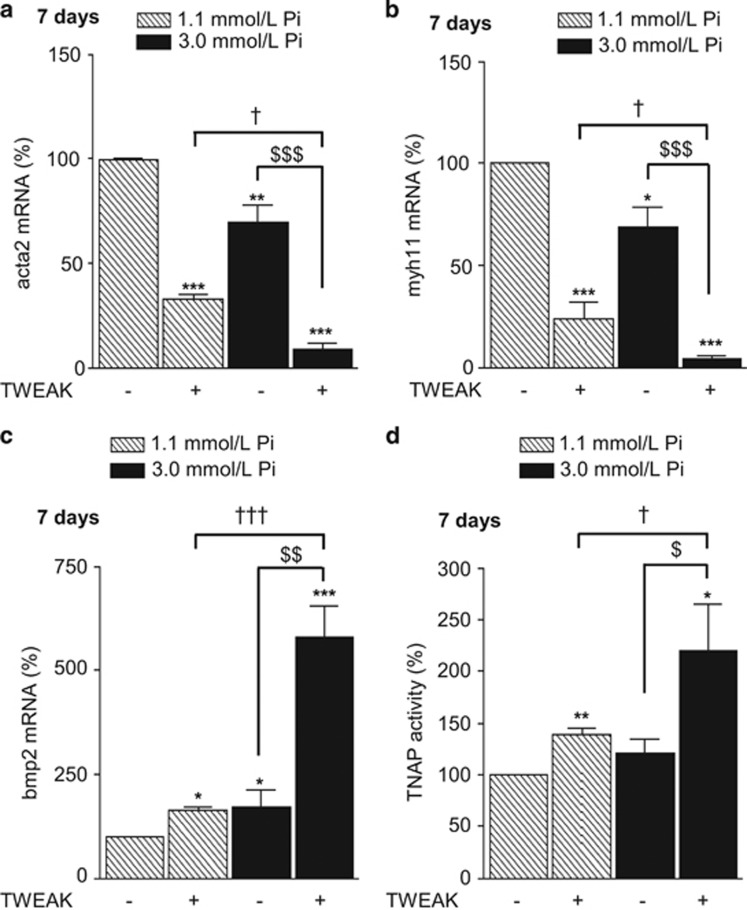
TWEAK/Fn14 promotes h-VSMCs osteogenic transition. The effects of TWEAK (100 ng/ml, 7 days) were assessed both in non-calcific (Ct: 1.1 mmol/l Pi) and pro-calcific (Pi: 3.0 mmol/l Pi) conditions. (**a**–**c**) Effects of TWEAK on acta2 (**a**), myh11 (**b**) and bmp2 (**c**) mRNA expression, assessed by quantitative reverse transcriptase-PCR. *P<0.05, **P<0.01, ***P<0.001 *vs* cells exposed to 1.1 mmol/l Pi without TWEAK. ^$^*P*<0.05, ^$$^*P*<0.01, ^$$$^*P*<0.001 *vs* cells exposed to 3.0 mmol/l Pi without TWEAK. ^†^*P*<0.05, ^†††^*P*<0.001 *vs* cells exposed to 1.1 mmol/l Pi with TWEAK. Results represent at least three independent experiments performed in triplicate. Error bars represent the S.E.M. (**d**) Effects of TWEAK on TNAP activity assessed by PNPP phosphatase assay. *P<0.05, **P<0.01 *vs* cells exposed to 1.1 mmol/l Pi without TWEAK. ^$^*P*<0.05 *vs* cells exposed to 3.0 mmol/l Pi without TWEAK. †*P*<0.05 *vs* cells exposed to 1.1 mmol/l Pi with TWEAK. Results represent five independent experiments performed in triplicate. Error bars represent the S.E.M.

**Figure 3:**
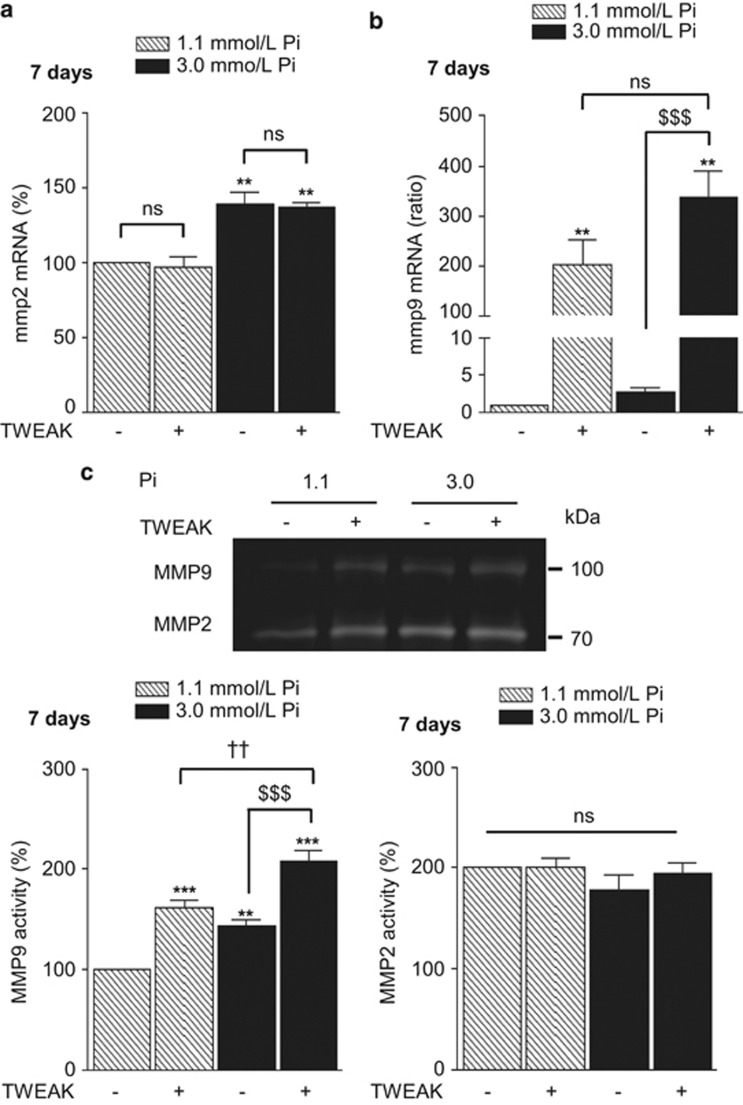
TWEAK/Fn14 promotes MMP9 but not MMP2 activity. (**a** and **b**) Effects of TWEAK on mmp2 (**a**) and mmp9 (**b**) mRNA expression assessed by quantitative reverse transcriptase-PCR. **P<0.01 *vs* cells exposed to 1.1 mM Pi without TWEAK. $$$*P*<0.001 *vs* cells exposed to 3.0 mmol/l Pi without TWEAK. Results represent at least three independent experiments performed in triplicate. Error bars represent the S.E.M. (**c**) Effects of TWEAK on MMP2 and MMP9 activity in h-VSMCs supernatants, assessed by gel zymography. **P<0.01, ***P<0.001 *vs* cells exposed to 1.1 mM Pi without TWEAK. ^$$$^*P*<0.001 *vs* cells exposed to 3.0 mmol/l Pi without TWEAK. ^††^*P*<0.01 *vs* cells exposed to 1.1 mmol/l Pi with TWEAK. Results represent at least three independent experiments performed in triplicate. Error bars represent the S.E.M. NS, not significant

To understand how the activation of TWEAK/Fn14 axis promoted Pi-induced h-VSMCs mineralization *in vitro*, the influence of TWEAK on h-VSMCs osteogenic transition, TNAP activity and matrix metalloproteinase (MMP) activity was evaluated in both non-calcific (1.1 mmol/l Pi) and pro-calcific (3.0 mmol/l Pi) conditions. Data obtained by qRT-PCR demonstrated that a 7-day exposure to 100 ng/ml TWEAK reduced by about 70% the expression of the contractile markers acta2 and myh11 in non-calcific conditions (Figures 2a and b) and significantly amplified by about three-fold Pi-induced decrease of both markers in pro-calcific environment (Figures 2a and b). Regarding h-VSMCs osteogenic conversion, TWEAK increased by 60% bmp2 mRNA expression in non-calcific condition and triggered a three-fold increase of bmp2 mRNA level in pro-calcific conditions (Figure 2c). TNAP hydrolyzes PPi, a strong inhibitor of calcium phosphate deposition, and thereby promotes calcification.^19^ In non- and pro-calcific conditions, 7-days of TWEAK increased by 25% TNAP expression (Supplementary Figure S4), which resulted in an increase of 40 and 120% of TNAP activity, respectively (Figure 2d). In the vasculature, MMP2 and MMP9 accelerate elastin degradation and promote calcification. In our model, TWEAK modulated neither MMP2 mRNA expression (Figure 3a) nor its activity (Figure 3c) but significantly increased both MMP9 mRNA expression (Figure 3b) and activity (Figure 3c) in non- and pro-calcific conditions.

In renal tubular cells, macrophages and kidney tissue, ERK engagement and canonical as well as non-canonical NF*κ*B activation contribute to TWEAK inflammatory effects.^10, 12, 20, 21^ In order to identify the intracellular pathways responsible for TWEAK/Fn14 pro-calcific effects, TWEAK signaling was blocked using small-molecule inhibitors or siRNA. As apoptosis is a key inducer of h-VSMCs calcification, we made sure that the inhibitor concentrations used to block TWEAK-induced h-VSMCs calcification did not modulate cell viability over the 7 days of treatment (Supplementary Figure S5A).

### TWEAK/Fn14-induced MAPK activation is not involved in TWEAK pro-calcific effects

In h-VSMCss, TWEAK favors ERK phosphorylation in a time-dependent manner, with a peak after 10 min of stimulation (Supplementary Figure S6A). Transfection with Fn14 siRNA totally abolished TWEAK-induced ERK phosphorylation as compared with scrambled transfected cells (Supplementary Figure S6B). Chronic exposure to 10 *μ*mol/l U0126, a selective inhibitor of MAPK kinases MEK1 and MEK2, inhibited TWEAK-induced ERK phosphorylation (Supplementary Figure S6C) but did not modulate TWEAK pro-calcific effects on Pi-induced h-VSMCs mineralization as compared with h-VSMCs not exposed to U0126 (Supplementary Figure S6D).

### TWEAK/Fn14-induced canonical NF*κ*B activation favors Pi-induced h-VSMCs calcification by promoting osteogenic transition, TNAP activity and MMP9 activity

**Figure 4:**
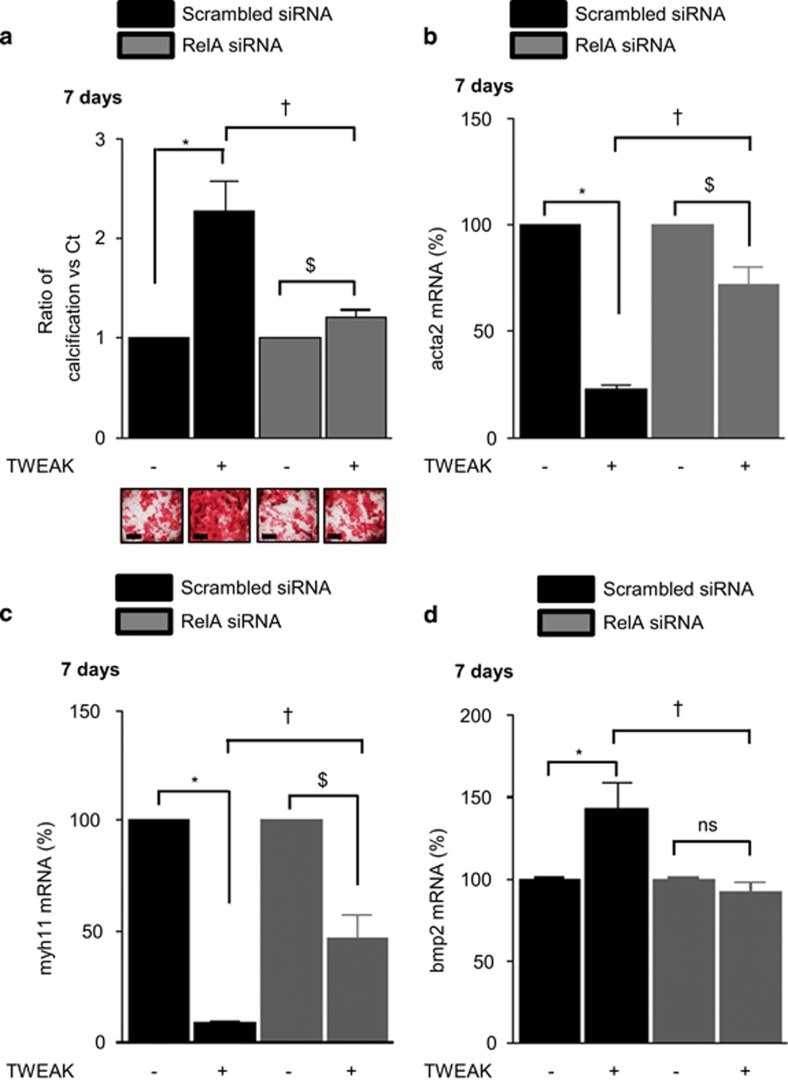
TWEAK/Fn14-induced canonical NF*κ*B activation favors Pi-induced h-VSMCs calcification by promoting osteogenic transition. h-VSMCss were transfected with either scrambled or RelA siRNA and exposed or not for 7 days to TWEAK in pro-calcific conditions (3.0 mmol/l Pi). (**a**) Impact of RelA downregulation on TWEAK/Fn14-induced h-VSMCs calcification in pro-calcific conditions (3.0 mmol/l Pi). Mineralization was measured by Alizarin red staining. Scale bars: 500μm. *P<0.05 vs scrambled transfected cells without TWEAK. ^$^*P*<0.05 vs RelA siRNA transfected cells without TWEAK. ^†^*P*<0.05 vs scrambled transfected cells exposed to TWEAK. Results represent three independent experiments performed in triplicate. Error bars represent the S.E.M. (**b**–**d**) Impact of RelA downregulation on TWEAK-induced modulation of acta2 (**b**), myh11 (**c**) and bmp2 (**d**) mRNA expression, assessed by quantitative reverse transcriptase-PCR. *P<0.05 vs scrambled transfected cells without TWEAK. $*P*<0.05 vs RelA siRNA transfected cells without TWEAK. ^†^*P*<0.05 vs scrambled transfected cells exposed to TWEAK. Results represent three independent experiments performed in triplicate. Error bars represent the S.E.M. NS, not significant

**Figure 5:**
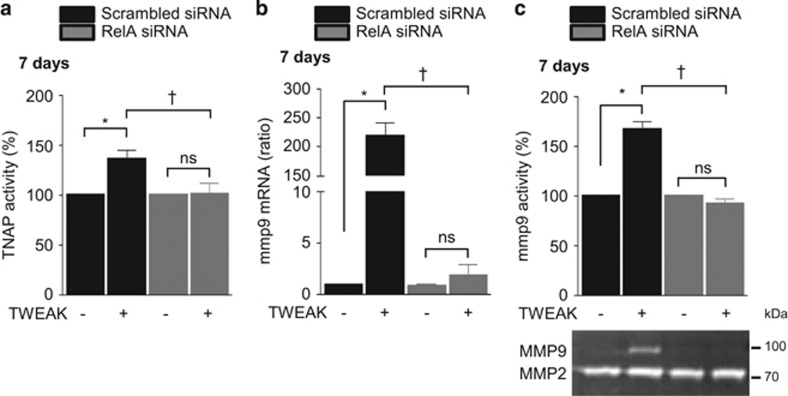
TWEAK/Fn14-induced canonical NF*κ*B activation favors TNAP and MMP9 activity. h-VSMCss were transfected with either scrambled or RelA siRNA and exposed or not for 7 days to TWEAK in pro-calcific conditions (3.0 mmol/l Pi). (**a**) Impact of RelA downregulation on TWEAK-modulation of TNAP activity. TNAP activity was assessed by pNPP phosphatase assay. *P<0.05 vs scrambled transfected cells without TWEAK. ^†^*P*<0.05 vs scrambled transfected cells exposed to TWEAK. Error bars represent the S.E.M. (**b**) Impact of RelA downregulation on TWEAK-induced mmp9 mRNA expression assessed by quantitative reverse transcriptase-PCR. *P<0.05 vs scrambled transfected cells without TWEAK. ^†^*P*<0.05 vs scrambled transfected cells exposed to TWEAK. Results represent three independent experiments performed in triplicate. Error bars represent the S.E.M. (**c**) Impact of RelA downregulation on TWEAK-induced MMP9 activity, assessed by gel zymography on cell supernatant. *P<0.05 vs scrambled transfected cells without TWEAK. ^†^*P*<0.05 vs scrambled transfected cells exposed to TWEAK. Results represent three independent experiments performed in triplicate. Error bars represent the S.E.M. NS, not significant

Phosphorylation and subsequent proteasomal degradation of I*κ*B is a hallmark of classical NF*κ*B pathway activation that facilitates the release of p65(RelA)/p50 dimers, which then migrate to the nucleus to modulate pro-inflammatory gene expression. Additional phosphorylation of RelA on Ser536 is essential for gene transactivation. In h-VSMCss, TWEAK induced RelA nuclear translocation and subsequent phosphorylation in a time-dependent manner, peaking at 10 min (Supplementary Figures S7A and C). Loss-of-function experiments using siRNA against Fn14 abolished TWEAK-induced RelA nuclear translocation and subsequent phosphorylation as compared with scrambled transfected cells (Supplementary Figures S7B and D). Transfection with a siRNA against RelA, which reduced by 75% RelA expression in h-VSMCss (Supplementary Figure S8A) and abolished RelA phosphorylation (Supplementary Figure S8B), significantly decreased by 80% TWEAK magnification of Pi-induced calcification as compared with scrambled transfected cells (Figure 4a). In pro-calcific conditions, RelA siRNA prevented by 60 and 50%, respectively, TWEAK-induced decrease of acta2 and myh11 mRNA expression (Figures 4b and c). Of interest, RelA targeting completely abolished TWEAK-induced increase in bmp2 mRNA expression (Figure 4d), TNAP activity (Figure 5a) and MMP9 mRNA expression (Figure 5b) and activity (Figure 5c) as compared with scrambled transfected cells.

### TWEAK/Fn14-induced non-canonical NF*κ*B activation promotes h-VSMCs loss of contractile phenotype as well as MMP9 activity but modulates neither bmp2 expression nor TNAP activity

**Figure 6:**
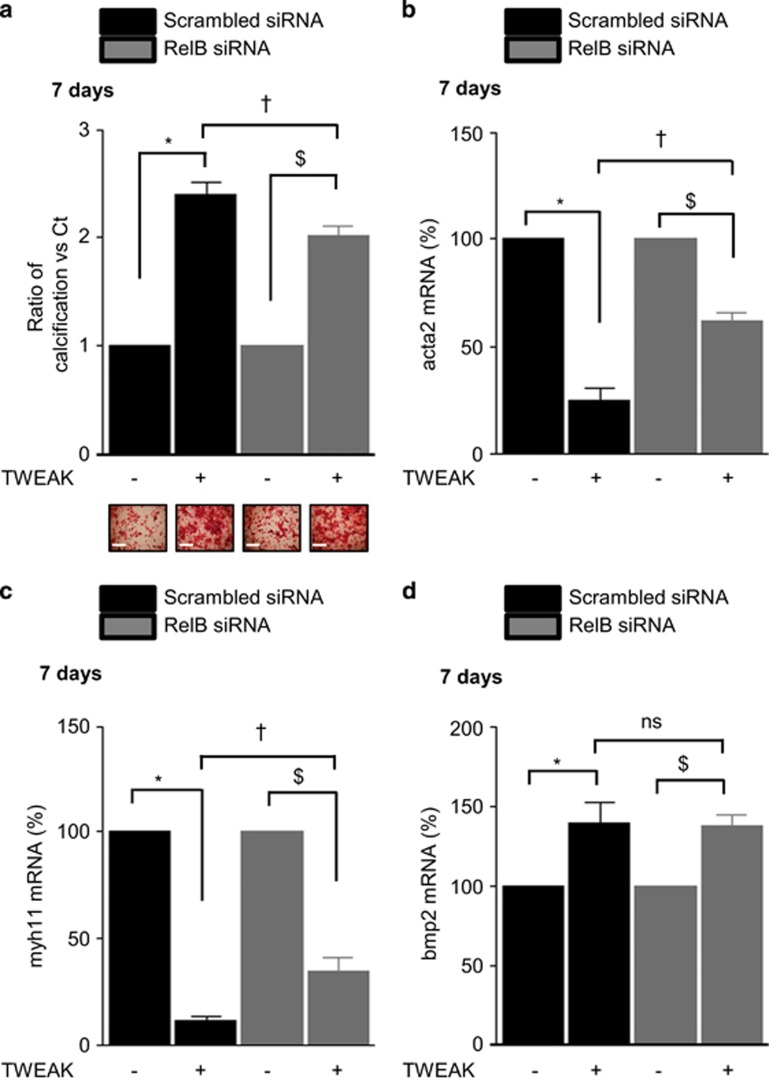
TWEAK/Fn14-induced non-canonical NF*κ*B activation promotes h-VSMCs loss of contractile phenotype but does not modulate bmp2 expression. h-VSMCss were transfected with either scrambled or RelB siRNA and exposed or not for 7 days to TWEAK in pro-calcific conditions (3.0 mmol/l Pi). (**a**) Impact of RelB downregulation on TWEAK/Fn14-induced h-VSMCs calcification. Mineralization was measured by Alizarin red staining. Scale bars: 500 *μ*m. *P<0.05 vs scrambled transfected cells without TWEAK. ^$^*P*<0.05 vs cells transfected with RelA siRNA and not exposed to TWEAK. ^†^*P*<0.05 vs scrambled transfected cells exposed to TWEAK. Results represent three independent experiments performed in triplicate. Error bars represent the S.E.M. (**b**–**d**) Impact of RelB downregulation on TWEAK-induced modulation of acta2 (**b**), myh11 (**c**) and bmp2 (**d**) mRNA expression, assessed by quantitative reverse transcriptase-PCR. *P<0.05 vs scrambled transfected cells without TWEAK. ^$^*P*<0.05 vs siRNA RelB transfected cells without TWEAK. ^†^*P*<0.05 vs scrambled transfected cells exposed to TWEAK. Results represent three independent experiments performed in triplicate. Error bars represent the S.E.M. NS, not significant

**Figure 7:**
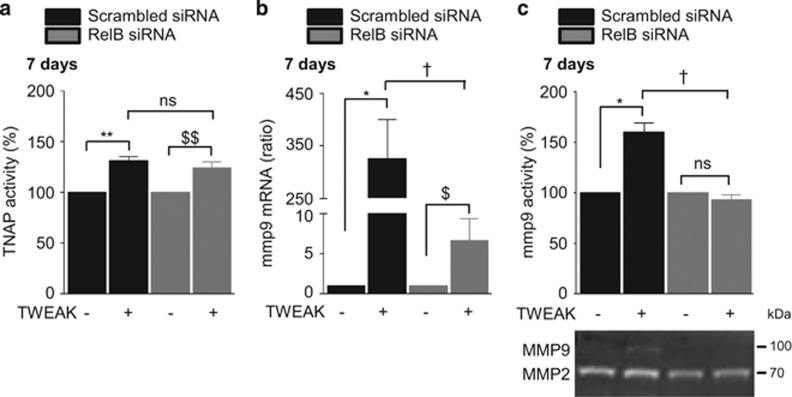
TWEAK/Fn14-induced non-canonical NF*κ*B activation does not influence TNAP activity but favors mmp9 activity. h-VSMCss were transfected with either scrambled or RelB siRNA and exposed or not for 7 days to TWEAK in pro-calcific conditions (3.0 mmol/l Pi). (**a**) Impact of RelB downregulation on TWEAK-induced modulation of TNAP activity. TNAP activity was assessed by pNPP phosphatase assay. **P<0.01 vs scrambled transfected cells without TWEAK. ^$$^*P*<0.05 vs cells transfected with siRNA RelB and not exposed to TWEAK. Error bars represent the S.E.M. (**b**) Impact of RelB downregulation on TWEAK-induced mmp9 mRNA expression assessed by quantitative reverse transcriptase-PCR. *P<0.05 vs scrambled transfected cells without TWEAK. ^$^*P*<0.05 vs siRNA RelB transfected cells without TWEAK. ^†^*P*<0.05 vs scrambled transfected cells exposed to TWEAK. Results represent three independent experiments performed in triplicate. Error bars represent the S.E.M. (**c**) Impact of RelB downregulation on TWEAK-induced MMP9 activity assessed by gel zymography in h-VSMCs supernatants. *P<0.05 vs scrambled transfected cells without TWEAK. ^†^*P*<0.05 vs scrambled transfected cells exposed to TWEAK. Results represent three independent experiments performed in triplicate. Error bars represent the S.E.M. NS, not significant

Activation of the non-canonical NF*κ*B pathway is characterized by NF*κ*B2 p100 processing to NF*κ*B p52. This results in the formation of p52/RelB heterodimers with transcriptional regulatory activity. In h-VSMCss, TWEAK decreased p100 and increased p52 in a time-dependent manner (Supplementary Figure S9A). However, as in other cell types, TNF-*α* did not activate the non-canonical NF*κ*B pathway (Supplementary Figure S10).^20^ Loss-of-function experiments using siRNA against Fn14 abolished TWEAK-induced p100 processing as compared with scrambled transfected cells (Supplementary Figure S9B). Transfection with a siRNA targeting RelB, which reduced by 80% RelB expression (Supplementary Figure S11A) and blocked TWEAK-induced expression of the non-canonical NF*κ*B target gene CCL21 (Supplementary Figure S11B), decreased by 20% the effects of TWEAK on Pi-induced calcification as compared with scrambled transfected cells (Figure 6a). In pro-calcific conditions, RelB downregulation prevented by 50 and 25%, respectively, TWEAK-induced downregulation of acta2 and myh11 mRNA expression (Figures 6b and c) but did not modulate TWEAK-induced bmp2 mRNA upregulation (Figure 6d) or TNAP activity (Figure 7a). Of interest, RelB targeting significantly reduced TWEAK-induced MMP9 mRNA expression (Figure 7b) and decreased MMP9 activity (Figure 7c).

### TWEAK upregulates Fn14 expression in h-VSMCss

Although Fn14 is upregulated *in vivo* in situations of cell or tissue stress, the mechanisms are poorly understood. Fn14 expression was upregulated after 7 days of exposure to TWEAK in both non- and pro-calcific conditions (Supplementary Figure S12A). The canonical, but not the non-canonical, activation of NF*κ*B was involved in Fn14 expression regulation in response to TWEAK (Supplementary Figure S12B).

## Discussion

The main finding is that the inflammatory cytokine TWEAK directly promotes h-VSMCs calcification through Fn14 engagement of both canonical and non-canonical NF*κ*B signaling. Chronic low-level systemic inflammation is common in aging, T2DM and CKD and associates with increased prevalence, severity and progression of VC and increased mortality.^22, 23, 24^ TWEAK promotes cardiovascular and kidney injury in experimental animal models.^25, 26, 27^ Reduced circulating sTWEAK levels have been associated with coronary artery disease,^28^ systolic heart failure,^29^ peripheral artery disease^30^ and aortic abdominal aneurysm^16^ and predict the severity of atherosclerosis.^31^ sTWEAK reduction is associated with a fast and high upregulation of TWEAK functional receptor Fn14,^14, 16^ which is thought to amplify sTWEAK local action within cardiovascular tissues. Circulating sTWEAK concentrations are decreased in end-stage renal disease and T2DM.^32^ However, higher sTWEAK levels are found in hemodialysis subjects with severe calcification than in those with nonsevere calcification^33^ and higher TWEAK levels are associated with a higher risk of all-cause and cardiovascular mortality,^34^ suggesting a link between sTWEAK and VC.

Although the role of TWEAK in atherosclerosis has been extensively characterized, no information is available concerning the role of TWEAK on medial calcification, a hallmark of CKD and T2DM. Our study demonstrated that TWEAK directly favors Pi-induced h-VSMCs calcification *in vitro* and supports a role of TWEAK in VC beyond favoring atherosclerosis. TWEAK amplified Pi-induced loss of contractile markers and acquisition of osteogenic markers, suggesting that TWEAK increases calcification *in vitro* by favoring Pi-induced h-VSMCs osteogenic transition. Furthermore, TWEAK increased TNAP expression and activity both in non- and pro-calcific conditions. TNAP hydrolyzes PPi, a VC inhibitor, and generates Pi, which is essential for hydroxyapatite formation. By degrading PPi, TNAP promotes calcification, changing the Pi/PPi ratio toward mineralization.^35^ Of interest, contrary to TNF-*α*,^36^ TWEAK did not induce h-VSMCs calcification in the absence of high phosphate medium (Supplementary Figure S1) even though it induced osteogenic transition, TNAP and MMP9 activity in non-calcific condition.

Many pro-calcific agents are known to promote h-VSMCs mineralization through the secretion of a pro-calcific matrix rich in type I collagen. In animal models, TWEAK promotes fibrosis.^13, 37^ In the present work, a 7-day exposure to TWEAK significantly reduced type I collagen expression both at the mRNA and protein level (Supplementary Figure S13), suggesting that TWEAK pro-calcific effects do not depend on the secretion of a pro-calcific matrix. These data are in accordance with a recent report from our group that showed decreased type I collagen expression in fibroblasts cultured *in vitro* in the presence of TWEAK.^13^ In this paper, the authors demonstrated that the pro-fibrotic effects of TWEAK observed *in vivo* result from TWEAK-induced fibroblast proliferation rather than collagen synthesis.

TWEAK binding to Fn14 triggers recruitment of TRAF2 and TRAF5, thus activating signaling pathways, such as MAPKs and NF*κ*B.^38, 39^ MAPK and NF*κ*B signaling had been implicated in TNF-*α*-induced VSMC calcification in high-phosphate medium.^40, 41^ In the present work, TWEAK binding to Fn14 induced MAPK signaling through ERK phosphorylation. However, inhibition of ERK1/2 phosphorylation did not influence TWEAK pro-calcific effects, underlining the differences in the mechanisms by which TNF-*α* and TWEAK favor VSMC calcification. In several cell types, TWEAK activates both canonical NF*κ*B signaling, a short-lived response resulting in nuclear migration of p65 (RelA)-containing complexes, and longer lasting non-canonical pathway resulting in nuclear migration of NF*κ*B2 p52/RelB-containing complexes.^20, 39, 42^ In our model, activation of Fn14 by TWEAK engaged both canonical and non-canonical NF*κ*B pathways. Blockade of canonical NFκB activation by siRNA significantly decreased TWEAK-induced h-VSMCs osteogenic transition and TNAP activity, which reduced by 80% its pro-calcific effects. These effects are similar to those of TNF-*α*, which promoted VSMC osteogenic transition and TNAP activity through canonical NF*κ*B activation.^43, 44, 45, 46^ Blocking the non-canonical NFκB pathway by a siRNA significantly decreased TWEAK-induced loss of h-VSMCs contractile phenotype but did not influence TWEAK-induced bmp2 expression or TNAP activity and reduced by 20% TWEAK pro-calcific effects. In this regard, TWEAK actions differ from those of TNF-*α*, as in our model, as in other cell types, TNF-*α* does not activate the non-canonical NF*κ*B pathway (Supplementary Figure S10).^20^ Furthermore, in our model Pi-induced calcification or TWEAK magnification of calcification did not depend on h-VSMCs apoptosis. By contrast, TNF-*α* was reported to favor Pi-induced VSMC apoptosis and subsequent calcification.^47^ In this regard, the Fn14 receptor lacks the death domain characteristic of the TNF receptor superfamily. Thus the molecular mechanisms of TWEAK-induced apoptosis appear to differ from those of TNF-*α*.

Vascular elastin degradation increases the extracellular matrix affinity for calcium,^48^ facilitating epitactic growth of hydroxyapatite along elastic lamellae. MMP2 and MMP9 have a crucial role in this process as MMP-2^−/−^ and MMP-9^−/−^ mice were resistant to elastin degradation and calcification.^49^ Pro-inflammatory cytokines, such as TNF-*α*^50^ and IL-1*β*,^51^ are strong inducers of MMP activity in the cardiovascular system. In the present work, long-term exposure to TWEAK favored the expression and activity of MMP9 but not of MMP2. These results are in accordance with a recent report showing a higher disruption of the elastic layer and increased MMP activity in the aortas from WT mice than in those from TWEAK^−/−^ or Fn14^−/−^ mice.^17^ In our model, h-VSMCss were cultured in a 2D system without elastin, so the pro-calcific effects of TWEAK observed *in vitro* may not be a direct consequence of an increased activity of MMP9. However, these data suggest that *in vivo* sTWEAK might increase vascular MMP9 activity and thus initiate VC in normal phosphate conditions or worsen VC triggered by hyperphosphatemia, as observed in CKD.

In healthy tissues, Fn14 expression is usually low or undetectable, although it is rapidly and highly upregulated under pathological conditions, such as myocardial infarction,^52^ restenosis after balloon injury^53^ or atherosclerosis.^14^ In cells from injured vascular walls,^14, 53, 54^ Fn14 is upregulated by cytokines, such as TNF-*α*, IL1-*β* and IFN-*γ*. In the present study, Fn14 expression was upregulated by TWEAK in both non- and pro-calcific conditions, which could amplify TWEAK pro-calcific properties. Little is known concerning the regulation of Fn14 expression, and only the RhoA/ROCK pathway has been related to Fn14 upregulation in cardiomyocytes and h-VSMCs.^14, 55^ For the first time to our knowledge, we report that the canonical, but not the non-canonical, activation of NF*κ*B promotes Fn14 expression in h-VSMCss exposed to TWEAK.

Previous report from our team demonstrated that TWEAK is expressed in both macrophages and smooth muscle cells within carotid atherosclerotic plaques.^14^ Confirming these data, in our model TWEAK is constitutively expressed by primary h-VSMCss (Supplementary Figure S14A). However, it was not modulated during the mineralization process neither by Pi nor by TWEAK itself (Supplementary Figure S14B), suggesting that in our model TWEAK pro-calcific effects are not related to increased autocrine TWEAK secretion in h-VSMCss.

In addition, TWEAK-induced NF*κ*B activation upregulates *in vivo* several cytokines implicated in inflammatory cell recruitment to injured vessel walls, such as MCP-1 and RANTES,^56^ which could magnify the direct pro-calcific effects. Besides, in cultured monocytes, TWEAK increases HMGB1,^21^ which binds both RAGE and TLR4 to locally mediate high glucose-induced VSMC calcification.^57^ Thus an *in vivo* contribution of TWEAK-induced HMGB1 to T2DM-related VC can be considered. Finally, TWEAK downregulates kidney Klotho expression and Klotho-deficient mice display VC.^58^

**Figure 8:**
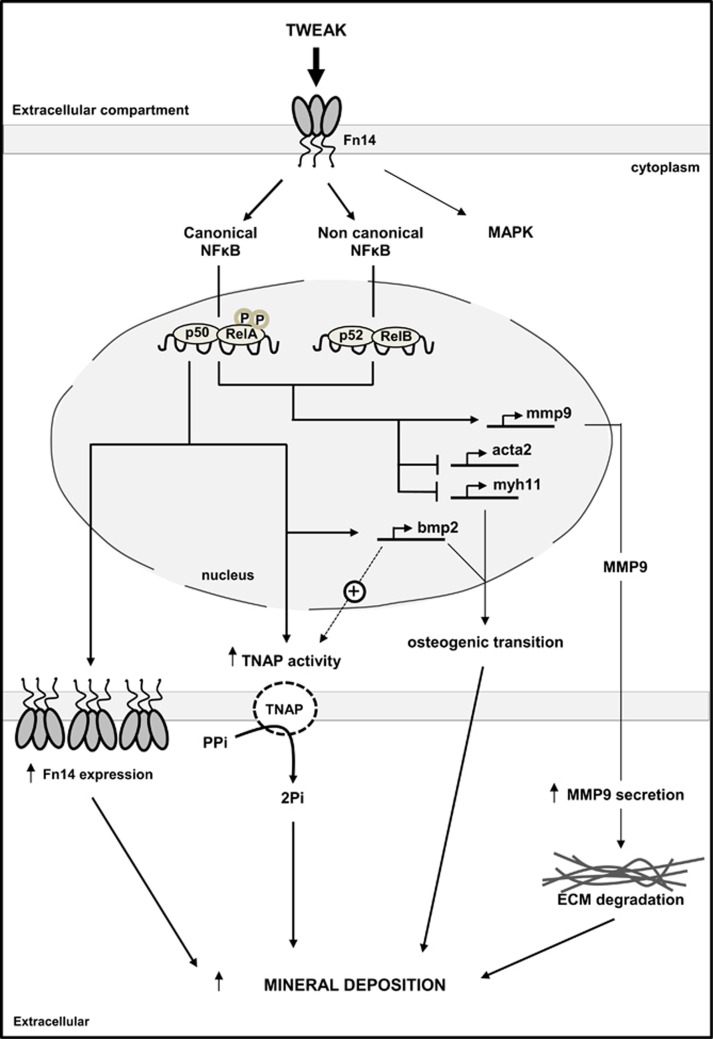
TWEAK/Fn14 pro-calcific effects on Pi-induced h-VSMCs calcification *in vitro*: a schematic representation. In pro-calcific conditions, TWEAK/Fn14 canonical NF*κ*B activation promoted h-VSMCs osteogenic transition, characterized by loss of h-VSMCs contractile markers acta2 and myh11 and upregulation of the osteogenic marker bmp2, a strong activator of TNAP. This was associated with an increase of TNAP activity, which degrades the calcification inhibitor PPi to release Pi that is essential for hydroxyapatite formation. TWEAK/Fn14-induced canonical NF*κ*B activation also increased Fn14 expression, which could amplify TWEAK pro-calcific properties. Both canonical and non-canonical NF*κ*B activation promoted MMP9 activity, which favors elastin degradation *in vivo*. TWEAK/Fn14-induced non-canonical NF*κ*B activation favors the loss of h-VSMCs contractile phenotype. By contrast, non-canonical NF*κ*B activation modulated neither bmp2 expression nor TNAP activity or Fn14 expression

In conclusion, TWEAK/Fn14-induced canonical NF*κ*B activation directly favors Pi-induced osteogenic transition and TNAP activity, promoting Pi-induced h-VSMCs calcification. Loss of h-VSMCs contractile markers as a consequence of TWEAK/Fn14-induced non-canonical NF*κ*B activation amplifies this phenomenon (Figure 8). To our knowledge, this is the first report of a direct pro-calcific effect of TWEAK and of the non-canonical NF*κ*B pathway involvement on medial calcification. In addition, TWEAK regulation of MMP activity and pro-inflammatory cytokine secretion, together with its pro-atherogenic properties make TWEAK a potential strong inducer of both intimal and medial calcification. Given the availability of neutralizing anti-TWEAK strategies, this work sheds light on TWEAK as a potential novel therapeutic target to prevent VC. Further studies *in vivo* on pathological animal models are currently under consideration in order to evaluate the consequences of neutralizing anti-TWEAK strategies on uremia-induced VC.

## Materials and Methods

### Human VSMCs (h-VSMCs)

H-VSMCs were isolated from lesion-free segments of proximal ascending aortas by a modified explant method.^59^ Samples were obtained after aortic valve surgery. Patients gave informed written consent in accordance with Spanish legislation (PROTOCOL EO34/2013). The study conforms with the Declaration of Helsinki.

### Mineralization assay

Mineralization of h-VSMCss (15000 cells/well, 24-well plates) was induced by exposure for 7 days to 1% FBS-supplemented DMEM containing 3.0mmol/l Pi (pro-calcific condition) or 1.1 mmol/l Pi (non-calcific condition). Increasing concentrations of recombinant human TWEAK (1–200ng/ml, Millipore, Bedford, MA, USA) were added to the culture medium and Pi-induced mineralization was assessed. Half of the culture medium was renewed every 2 days. Detection and quantification of mineral deposition were performed using Alizarin Red staining. Briefly, samples were fixed (50% ethanol 5 min, 95% ethanol 5 min), stained for 5 min with Alizarin red S (40 mmol/l, pH 4.2), rinsed with 50% ethanol, and photographed. Alizarin red was then solubilized by incubation in a buffer containing 10 mmol/l NaH_2_PO_3_ and 10% hexadecylpyridinium chloride monohydrate prior to reading at 570 nm. When mineralization was assessed in transfected h-VSMCss, transfections were performed 24 h before and 96 h after the first exposure to the treatment of interest. For experiments using 10 *μ*mol/l U0126, the inhibitor was added every 2 days with fresh media.

### h-VSMCs transfection

h-VSMCs were transfected with siRNA diluted in OptiMEM (Gibco, Carlsbad, CA, USA), using lipofectamine (Invitrogen, Paisley, UK). siRNA targeting Fn14, RelA and RelB (Invitrogen) were used at a concentration of 10, 50 and 20 nM respectively. Scrambled siRNA (Ambion, Foster City, CA, USA) served as negative control.

### qRT-PCR

Total RNA was extracted using the TRI reagent method (Sigma, St Louis, MO, USA). Then 1 *μ*g RNA was reversed transcribed into cDNA using the ‘High Capacity cDNA Reverse Transcription Kit' (Applied Biosystems, Foster City, CA, USA) according to the manufacturer's instructions. Predeveloped primer for acta2, myh11, bmp2, spp1, mmp2, mmp9, tnfsf12 and GAPDH were from Applied Biosystems. Quantitative PCR was performed by 7500 Real Time PCR System with the Prism 7000 System SDS Software (Applied Biosystems) and RNA expression of the different genes was corrected for GAPDH.

### Western blotting

H-VSMCs (60 000 cells/well in six-well plates) were stimulated with Pi and/or TWEAK. Samples were then homogenized in lysis buffer (50 mM Tris-HCl, 150 mM NaCl, 2 mM EDTA, 2 mM EGTA, 0.2% Triton X-100, 0.3% NP-40, 0.1 mM PMSF and 1 *μ*g/ml pepstatin A) and separated by 10–12% SDS-PAGE under reducing conditions. After electrophoresis, samples were transferred to PVDF membranes (Millipore), blocked with 5% skimmed milk in PBS/0.5% v/v Tween 20 for 1 h, washed with PBS/Tween and incubated overnight at 4 °C with mouse monoclonal anti-ERK phosphorylated (1 : 250), rabbit polyclonal anti-ERK2 (1/250), rabbit polyclonal anti-p65 (RelA) (1/500), rabbit polyclonal anti-RelB (1/500) (Santa Cruz Biotechnology, Santa Cruz, CA, USA), rabbit monoclonal anti-phospho-NF*κ*B p65 (1 : 500), rabbit polyclonal anti-Fn14 (1 : 1000), rabbit polyclonal anti-NF*κ*B p52/p100 (1 : 1000) (Cell Signaling, Danvers, MA, USA) or rabbit polyclonal anti-TNAP (1 : 500) (Abcam, Cambridge, UK). Antibodies were diluted in 5% milk PBS/Tween. Blots were washed with PBS/Tween and incubated with appropriate horseradish peroxidase-conjugated secondary antibody (1 : 2000, Amersham, Aylesbury, UK). After washing with PBS/Tween, blots were developed with the chemiluminescence method (ECL) (Amersham, Aylesbury, UK) and then probed with mouse monoclonal anti-*α*-tubulin antibody (1 : 2000, Sigma) or mouse monoclonal anti-GAPDH antibody (1 : 3000, Millipore) following the same method and the levels of expression were corrected for minor differences in loading.

### Confocal microscopy

H-VSMC monolayers on coverslips were fixed in 4% paraformaldehyde for 10 min and quenched in 100 mmol/l glycin/PBS for additional 10 min. Cells were then permeabilized in 0.2% Triton X-100/PBS for 10 min. Non-specific binding was blocked by incubation in 1% BSA/PBS for 30 min. Later, h-VSMCss were incubated with rabbit polyclonal anti-RelA (1 : 75), mouse monoclonal anti-MYH11 (1 : 50) or rabbit polyclonal anti-TWEAK (1 : 100) (Santa Cruz Biotechnology) for 1 h, followed by 1 h of FITC secondary antibody (1 : 200, Sigma). Nuclei were counterstained with propidium iodide. Coverslips were mounted on glass microscope slides prior to fluorescent detection.

### TNAP activity

h-VSMCs (60000 cells/well, six-well plates) were starved overnight in 1% FBS-supplemented DMEM prior to a 7-day exposure to 1.5 ml 1% FBS-supplemented DMEM containing or not 3 mmol/l Pi and/or TWEAK. In all, 750 *μ*l fresh medium was added every 2 days. TNAP activity was measured with the pNPP Phosphatase Assay Kit (BioAssay Systems, Hayward, CA, USA) as described previously.^60^

### Gel zymography

H-VSMCs were cultured as for TNAP activity. After 7 days, h-VSMCs supernatants were centrifuged to remove debris and were diluted by two for assessment of MMP2 activity. Samples were concentrated 10-fold with a 10-kDa microcon (Millipore) for detection of MMP9 activity. MMP activity was assessed by in-gel gelatinase zymogram (Zymogran gels, Life Technologies, Foster City, CA, USA) as described previously.^17^

### Statistical analysis

Results represent at least three independent experiments performed in triplicate using cells isolated from different donors. Results are expressed as mean±S.E.M. Differences between groups were evaluated using one-way ANOVA with Tukey's *post-hoc* tests using the Prism software (Graphpad, Version 5.0 GraphPad Software Inc., San Diego, CA, USA). For pairs of parameters, data were analyzed using non-parametric Mann–Whitney test. A *P*-value <0.05 was considered statistically significant.

## Abbreviations

BMP2: bone morphogenetic protein 2
CKD: chronic kidney disease
Fn14: fibroblast growth factor-inducible molecule 14
H-VSMC: human vascular smooth muscle cell
MAPK: mitogen-activated protein kinase
MMP: matrix metalloproteinase
PPi: pyrophosphate
T2DM: type 2 diabetes mellitus
TNAP: tissue non-specific alkaline phosphatase
sTWEAK: soluble TWEAK
TWEAK: TNF-like weak inducer of apoptosis
VC: vascular calcification
