# Supplementary material for: TWEAK favors phosphate-induced calcification of vascular smooth muscle cells through canonical and non-canonical activation of NFκB
Source: Cell Death Dis. 2016 Jul 21;7(7):e2305–. doi: 10.1038/cddis.2016.220 (PMC4973358; doi:10.1038/cddis.2016.220)
Supplement: Supplementary Figures [file cddis2016220x1.pdf]

Supplemental Figures Summary

Supplemental Figure 1 shows that TWEAK does not induce h-VSMCs calcification in non-calcific conditions.

Supplemental Figure 2 gathered data from MTT (A.), cell cycle analysis (B.), DAPI staining (C.), Annexin/7ADD (D.) and caspase 3 activity (E.) demonstrating that in our model TWEAK and phosphate do not influence cell survival, mitosis or apoptosis.

Supplemental Figure 3 shows the controls of transfection performed to check the functionality of Fn14 siRNA.

Supplemental Figure 4 shows that TWEAK-induced increase in TNAP activity detailed in Figure 2.D is associated with an increase in TNAP expression.

Supplemental Figure 5 presents data from MTT test demonstrating that neither U0126 nor siRNAs modulate h-VSMCs viability during the mineralization process.

Supplemental Figure 6 shows that TWEAK activation of Fn14 induces MAPK signaling in h-VSMCs. This signaling is not involved in TWEAK pro-calcific effects.

Supplemental Figure 7 demonstrates that TWEAK activation of Fn14 activates the canonical NFκB signaling in h-VSMCs.

Supplemental Figure 8 presents the functionality of Rel A siRNA to downregulate Rel A expression and to block TWEAK-induced Rel A phosphorylation.

Supplemental Figure 9 demonstrates that TWEAK activation of Fn14 activates the non-canonical NFκB signaling in h-VSMCs.

Supplemental Figure 10 demonstrates that contrary to TWEAK, TNF-α does not activate non-canonical NFκB signaling in h-VSMCs.

Supplemental Figure 11 presents the functionality of Rel B siRNA to downregulate Rel B expression and to block TWEAK-induced protein expression of the non-canonical NFκB target gene CCL21.

Supplemental Figure 12 makes the demonstration that TWEAK promotes Fn14 expression through canonical and non-canonical activation of NFκB signaling in h-VSMCs.

Supplemental Figure 13 shows that TWEAK reduces collagen expression both at mRNA and protein levels in h-VSMCs during the mineralization process.

Supplemental Figure 14 shows that TWEAK is expressed in h-VSMCs in basal culture conditions and that its expression is not modulated during the mineralization process.

Supplemental Figures

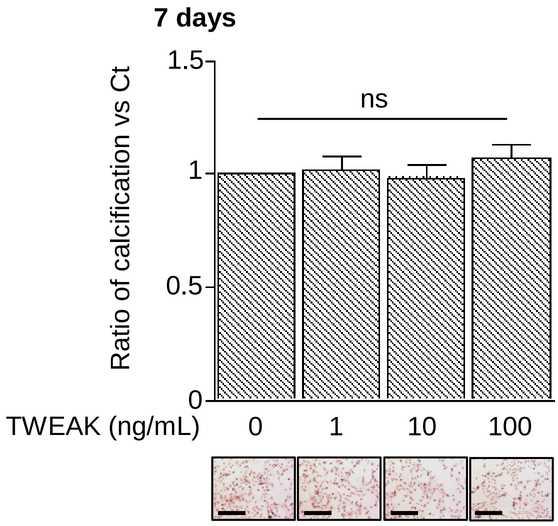

Supplemental figure 1. *TWEAK does not induce h-VSMCs calcification in non-calcific conditions.* H-VSMCs were cultured for 7 days in non-calcific conditions (1.1 mmol/L Pi) in presence or absence of TWEAK (1-100 ng/mL) in medium containing 1% FBS. Scale bars: 500µm. Results represent four independent experiments. Error bars represent the S.E.M.

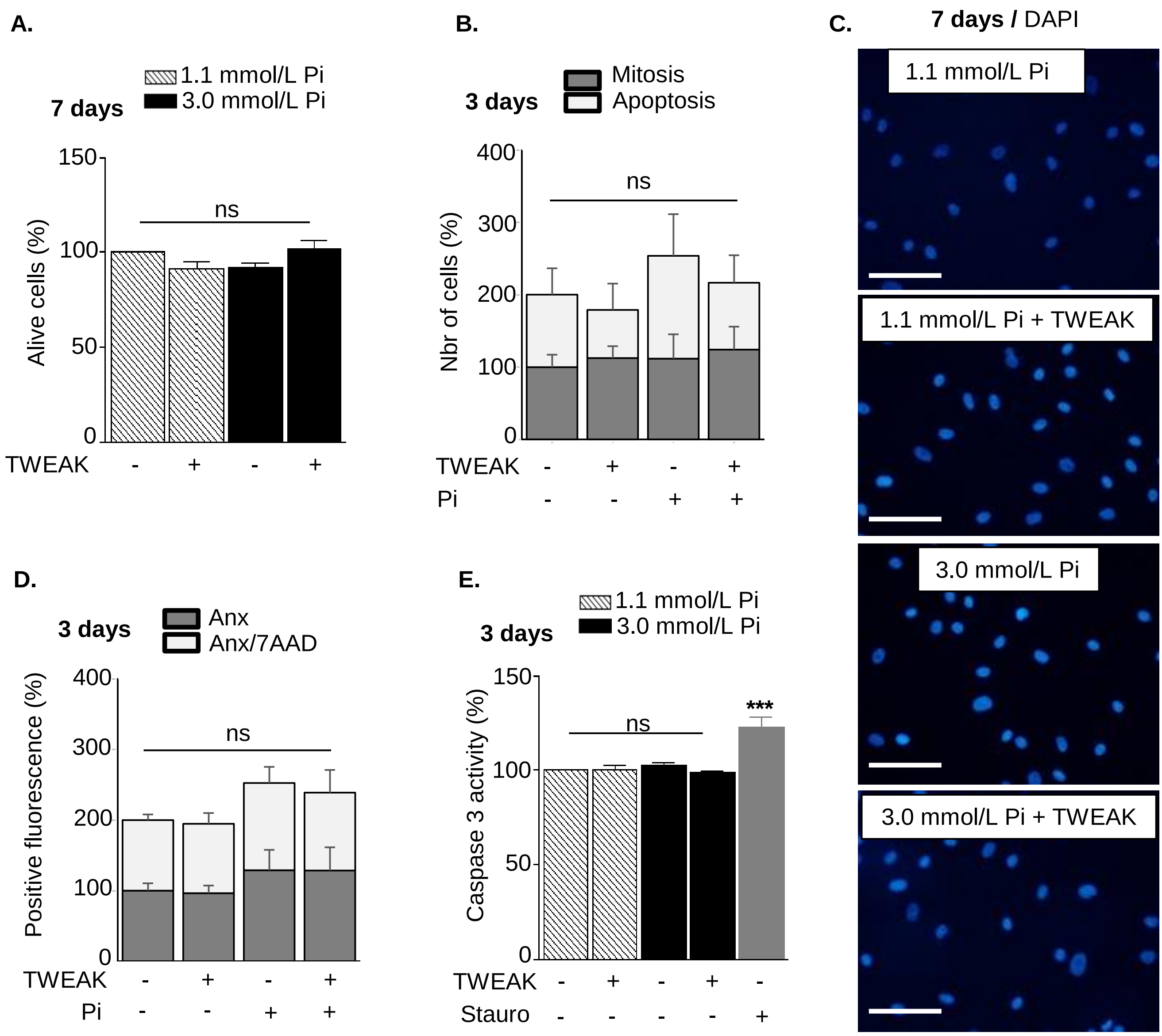

Supplemental figure 2. *Neither TWEAK nor Pi modulate h-VSMCs proliferation and survival*. H-VSMCs were cultured for 3 days or 7 days in non-calcific (1.1 mmol/L Pi) or pro-calcific conditions (3.0 mmol/L Pi) in presence or absence of 100 ng/mL TWEAK in medium containing 1% FBS. Stauroporine was used as a positive control for cell death to validate the functionality of every experiment (data not shown). **A.** Effects of a 7-day exposure to TWEAK on cell survival assessed by the MTT assay. Results represent three independent experiments performed in triplicate. Error bars represent the S.E.M. **B.** Effects of a 3-day exposure to TWEAK on h-VSMCs mitosis and apoptosis. Briefly, cells were permeabilized and stained with propidium iodide, and the percentage of apoptotic cells (with decreased DNA staining) and proliferating cells (with increased DNA content) was counted

by flow cytometry. Results represent three independent experiments performed in triplicate. Error bars represent the S.E.M. **C.** Effects of a 7-day exposure to TWEAK on h-VSMCs apoptosis assessed morphologically by a DAPI staining. Pictures represent three independent experiments performed in triplicate. Images scale bars: 100µm. **D.** Effects of a 3-day exposure to TWEAK on h-VSMCs early apoptosis and cell death assessed by the PE Annexin V apoptosis detection kit. Results represent five independent experiments performed in triplicate. Error bars represent the S.E.M. **E.** Effects of a 3-day exposure to TWEAK on Caspase 3 activity. *** $p<0.001$ vs. cells exposed to1.1 mmol/L Pi without TWEAK. Results represent three independent experiments performed in triplicate. Error bars represent the S.E.M.

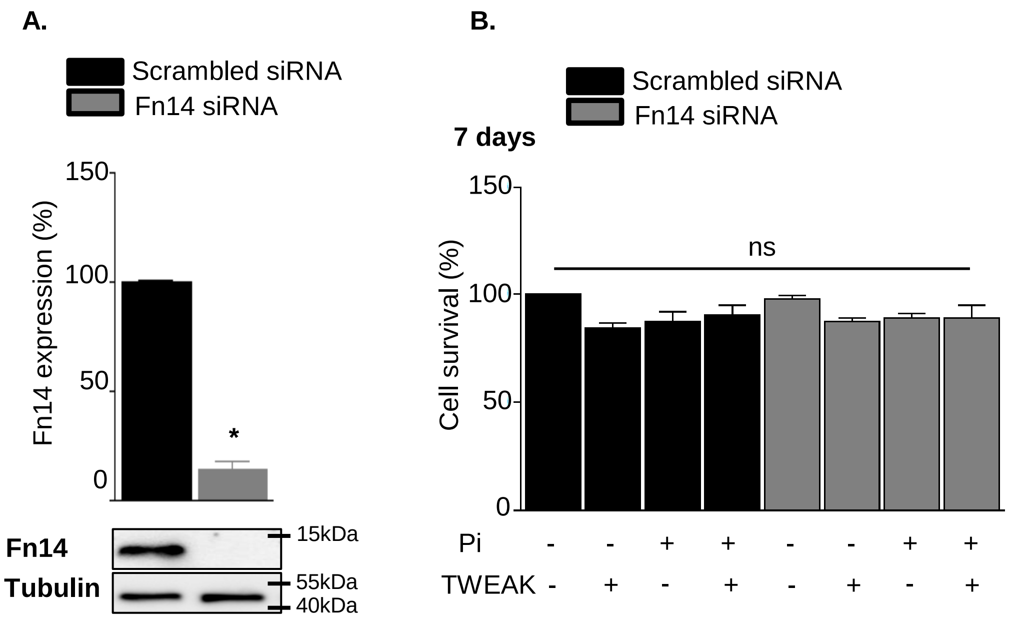

Supplemental figure 3. *Effects of Fn14 siRNA on Fn14 expression and h-VSMCs viability.* **A.** Evaluation of the efficiency of Fn14 siRNA to down-regulate Fn14 protein expression (western blot). * $p<0.05$ vs scrambled transfected cells. Results represent three independent experiments. Error bars represent the S.E.M. **B.** Impact of the transfection with a siRNA targeting Fn14 on h-VSMCs viability assessed by MTT. Transfected H-VSMCs were cultured for 7 days in non-calcific (1.1 mmol/L Pi) or pro-calcific conditions (3.0 mmol/L Pi) in presence or absence of 100 ng/mL TWEAK. Results represent three independent experiments. Error bars represent the S.E.M.

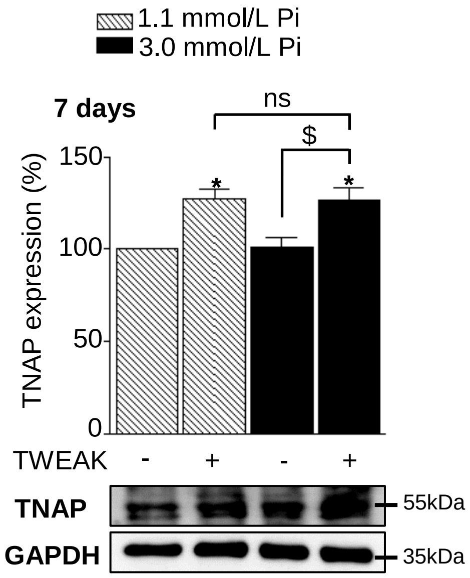

Supplemental figure 4. *TWEAK/Fn14 promotes TNAP expression in h-VSMCs.* The effects of TWEAK (100ng/mL, 7 days) were assessed both in non-calcific (Ct: 1,1 mmol/L Pi) and pro-calcific (Pi: 3.0 mmol/L Pi) conditions. TNAP expression was assessed by western blot. * $p<0.05$ vs. cells exposed to 1.1 mmol/L Pi without TWEAK. $ $p<0.05$ vs. cells exposed to 3.0 mmol/L Pi without TWEAK. Results represent five independent experiments performed in triplicate**.** Error bars represent the S.E.M.

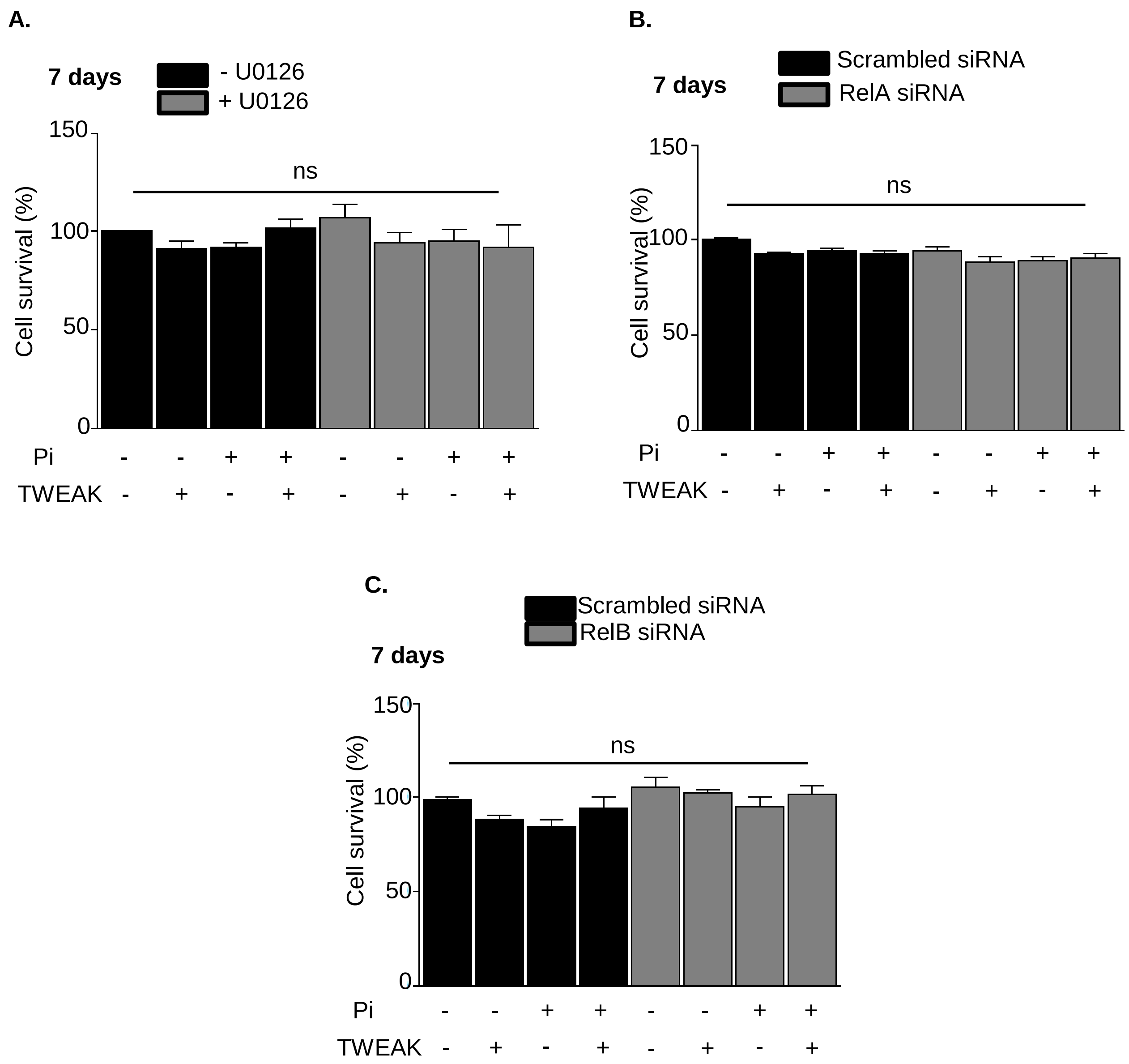

Supplemental figure 5. Neither U0126 nor siRNAs modulate *h-VSMCs viability during the mineralization process.* H-VSMCs, transfected or not with either a scrambled siRNA or a siRNA targeting RelA or RelB were cultured for 7 days in non-calcific (1.1 mmol/L Pi) or pro-calcific conditions (3.0 mmol/L Pi) in presence or absence of 100 ng/mL TWEAK and / or the MAPK inhibitor U0126. **A.** Impact of U0126 on h-VSMCs viability assessed by MTT. Error bars represent the S.E.M. **B.** and **C.** Impact of the transfection with a siRNA targeting RelA (B.) and RelB (C.) on h-VSMCs viability assessed by MTT. Results represent three independent experiments. Error bars represent the S.E.M.

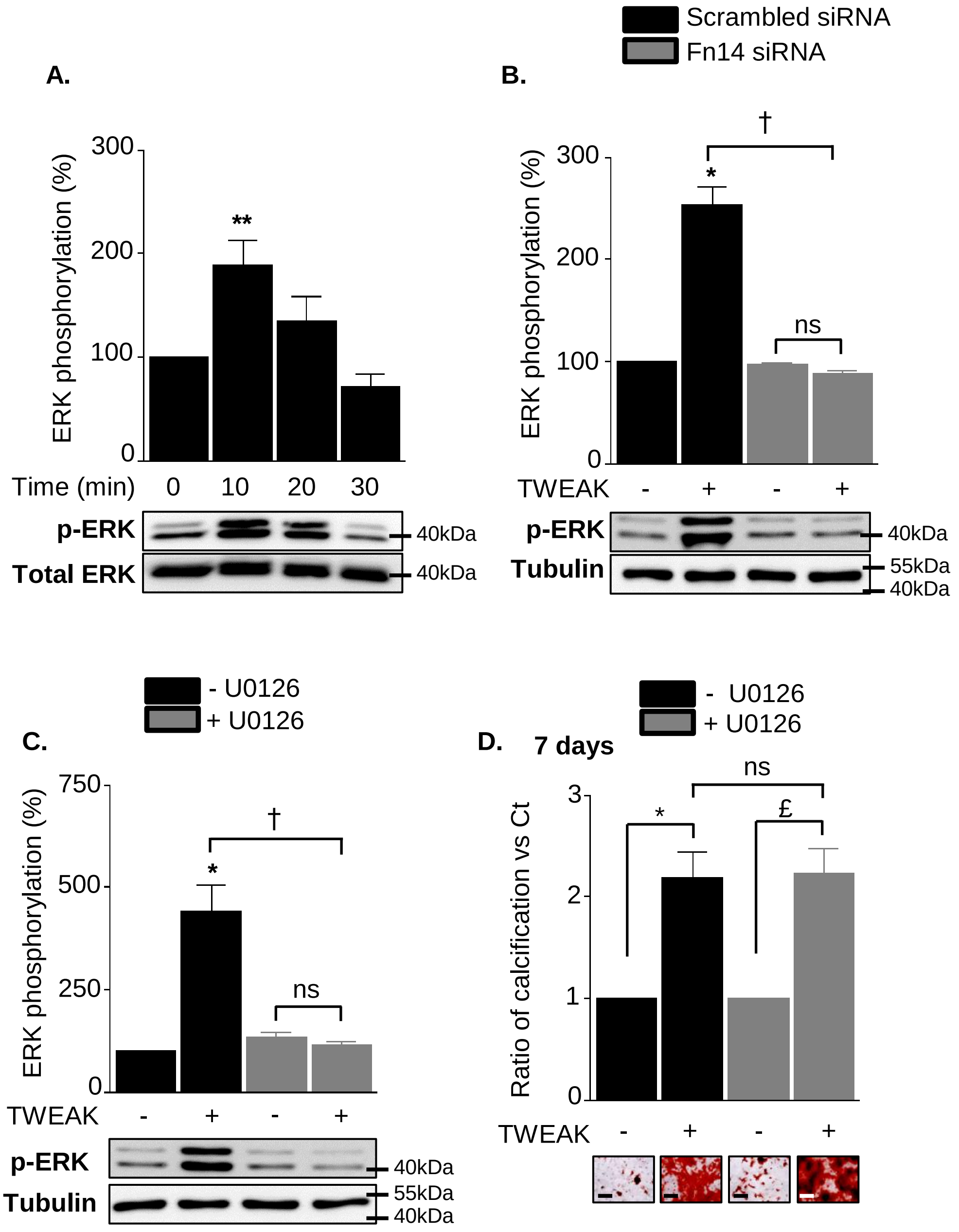

Supplemental figure 6. *TWEAK/Fn14-induced MAPK activation is not involved in TWEAK pro-calcific effects.* **A.** Immunoblotting of total ERK and p-ERK performed on cell lysates prepared from h-VSMCs stimulated with 100 ng/mL of TWEAK for 10, 20 and 30 min. ** p<0.01 vs baseline control condition. Results represent five independent experiments performed in triplicate. Error bars represent the S.E.M. **B.** Immunoblotting of p-ERK and tubulin performed on cell lysates prepared from h-VSMCs transfected with either scrambled or Fn14 siRNA, and exposed for 10 min to 100 ng/mL of TWEAK. * p<0.05 vs scrambled transfected cells without TWEAK. † p<0.05 vs. scrambled transfected cells exposed to TWEAK. Results represent four independent experiments. Error bars

represent the S.E.M. **C.** Efficiency of U0126 to block TWEAK/Fn14-induced ERK phosphorylation. Immunoblotting of p-ERK and tubulin performed on cell lysates prepared from h-VSMCs exposed or not for 1 h to U0126 and stimulated with TWEAK for 10 min. * $p<0.05$ vs control condition (unstimulated cells). † $p<0.05$ cells exposed to TWEAK in absence of U0126. Results represent three independent experiments. Error bars represent the S.E.M. **D.** Impact of U0126 on TWEAK/Fn14-induced h-VSMCs calcification in pro-calcific conditions (3.0 mmol/L Pi), assessed by the alizarin red staining method. Images scale bar: 500µm. * $p<0.05$ vs control condition (unstimulated cells). $ $p<0.05$ vs cells exposed to U0126 without TWEAK. Results represent three independent experiments performed in triplicate. Error bars represent the S.E.M.

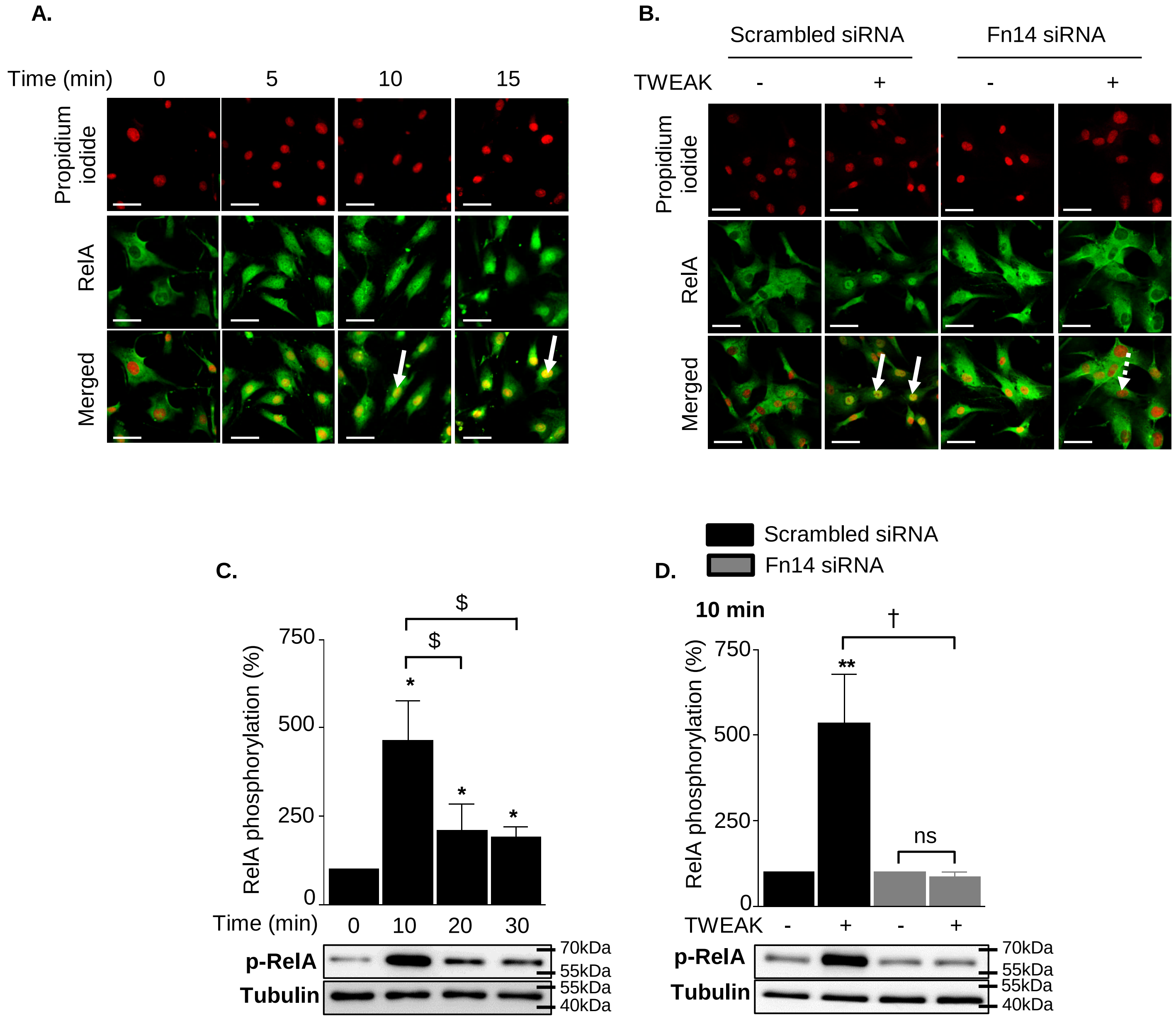

Supplemental figure 7. *TWEAK/Fn14 activates canonical NFκB signaling in h-VSMCs.* **A.** and **B.** Study of RelA nuclear translocation assessed by confocal microscopy after RelA immunostaining (green) counterstained with propidium iodide (PI, red). **A.** Effects of TWEAK on RelA nuclear translocation. TWEAK-induced RelA nuclear translocation appeared in yellow within 5-15 minutes (arrows). Images scale bars: 50µm. Results represent three independent experiments. **B.** Impact of Fn14 downregulation on TWEAK-induced RelA nuclear translocation. The study was performed on h-VSMCs transfected with either scrambled or Fn14 siRNA and stimulated for 15 min with 100 ng/mL of TWEAK. Continuous arrows denote nuclear RelA translocation (yellow) in response to TWEAK exposure in scrambled transfected h-VSMCs, while dotted lined arrows denote lack of RelA

translocation in Fn14 transfected h-VSMCs. Images scale bars: 50µm. **C.** Immunoblotting of p-RelA and tubulin performed on cell lysates prepared from h-VSMCs stimulated with TWEAK for 10, 20 and 30 min. * $p<0.05$ vs baseline control condition. $ $p<0.05$ vs. cells exposed to TWEAK for 10 min. Results represent four independent experiments. Error bars represent the S.E.M. **D.** Immunoblotting of p-RelA and tubulin performed on cell lysates prepared from h-VSMCs transfected with either scrambled or Fn14 siRNA and stimulated for 10 min with 100 ng/mL of TWEAK. ** $p<0.01$ vs scrambled transfected cells without TWEAK. † $p<0.05$ vs. scrambled transfected cells exposed to TWEAK. Results represent five independent experiments. Error bars represent the S.E.M.

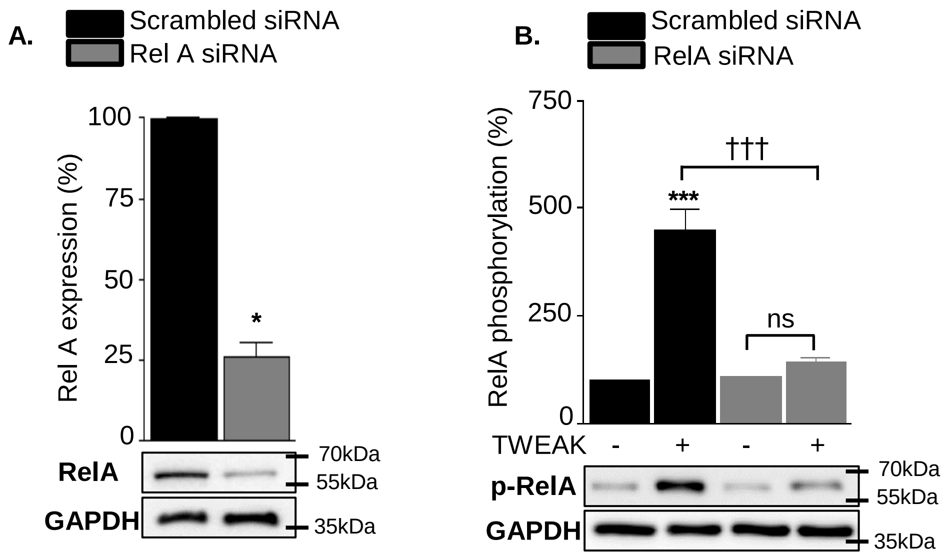

Supplemental figure 8. *Effects of RelA siRNA on RelA expression and phosphorylation in h-VSMCs.* **A.** Efficiency RelA siRNA to downregulate RelA expression. Immunoblotting of RelA and GAPDH performed on cell lysates prepared from h-VSMCs transfected with either scrambled or RelA siRNA. * p<0.05 vs scrambled transfected cells. Results represent three independent experiments. Error bars represent the S.E.M. **B.** Efficiency RelA siRNA to block TWEAK/Fn14-induced RelA phosphorylation. Immunoblotting of p-RelA and GAPDH performed on cell lysates prepared from h-VSMCs transfected with either scrambled or RelA siRNA and exposed or not to 100ng/mL of TWEAK for 10 min. *** p<0.001 vs scrambled transfected cells not exposed to TWEAK. ††† p<0.001 vs scrambled transfected cells treated with TWEAK. Results represent three independent experiments. Error bars represent the S.E.M.

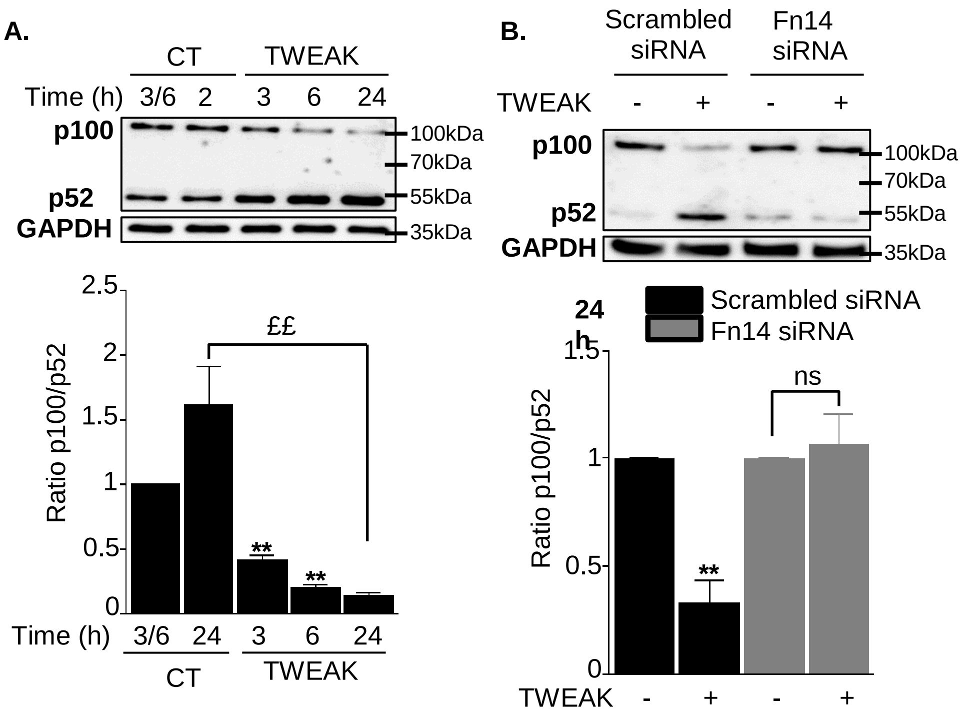

Supplemental figure 9. *TWEAK/Fn14 Induces non-canonical activation of NFκB pathway in h-VSMCs.* **A.** Immunoblotting of NFκB2 p100, p52 and GAPDH performed on cell lysates prepared from h-VSMCs stimulated with 100ng/mL TWEAK for 3, 6 and 24 hours. ** p<0.01 vs control condition (without TWEAK for 3 and 6 hours). ££ p<0.01 vs. control condition (without TWEAK for 24 hours). Results represent five independent experiments. Error bars represent the S.E.M. **B.** Immunoblotting of NFκB2 p100, p52 and GAPDH performed on cell lysates prepared from h-VSMCs transfected with either scrambled or Fn14 siRNA, and stimulated with TWEAK for 6 hours. ** p<0.01 vs scrambled transfected cells without TWEAK. Results represent six independent experiments. Error bars represent the S.E.M.

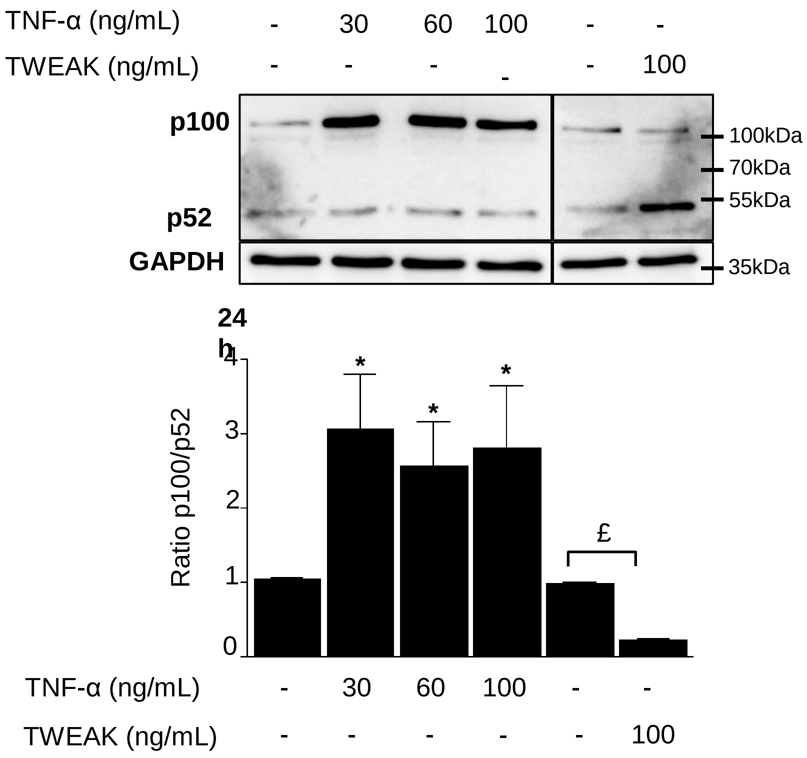

Supplemental figure 10. *TNF-α does not activate non-canonical NFκB signaling in h-VSMCs.* Immunoblotting of NFκB2 p100, p52 and GAPDH performed on cell lysates prepared from h-VSMCs exposed for 24 hours to increasing concentrations of TNF-α (0, 30, 60 or 100 ng/mL). TNF-α did not promote NFκB2 p100 processing into NFκB2 p52. TWEAK (100ng/mL, 24 hours) was used as a positive stimulus for p100 processing into p52. * $p<0.05$ cells exposed to TNF-α vs. untreated cells. £ $p<0.05$ cells exposed to TWEAK vs. untreated cells. Results represent four independent experiments. Error bars represent the S.E.M.

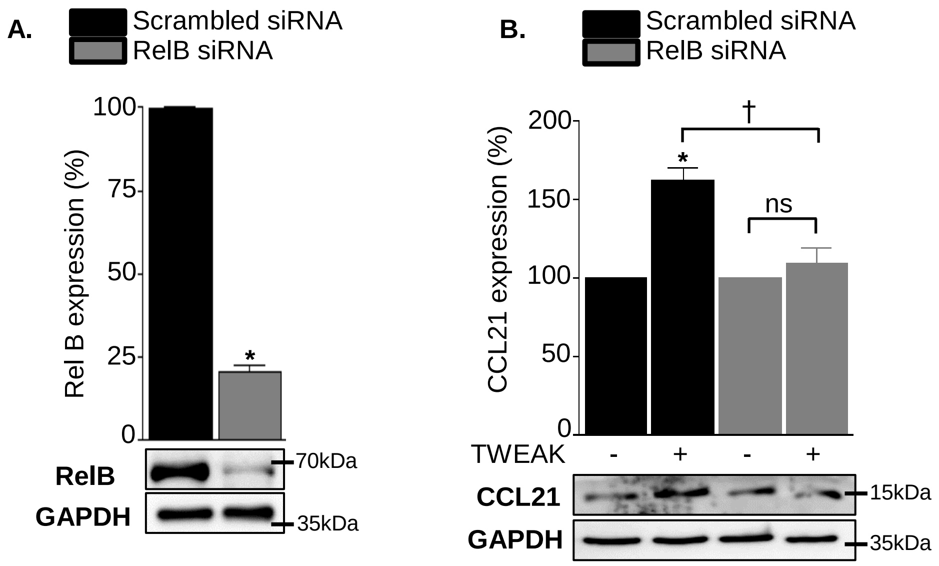

Supplemental figure 11. *Effects of RelB siRNA on RelB expression and* TWEAK/Fn14-induced *CCL21 expression in h-VSMCs.* **A.** Efficiency of RelB siRNA to downregulate RelB expression. Immunoblotting of RelB and GAPDH performed on cell lysates prepared from h-VSMCs transfected with either scrambled or RelB siRNA. * $p<0.05$ vs scrambled transfected cells. Error bars represent the S.E.M. **B.** Efficiency of RelB siRNA to block TWEAK/Fn14-induced CCL21 protein expression. Immunoblotting of CCL21 and GAPDH performed on cell lysates prepared from h-VSMCs transfected with either scrambled or RelB siRNA and exposed or not to 100 ng/mL of TWEAK for 24 hours. * $p<0.05$ vs scrambled transfected cells not exposed to TWEAK. † $p<0.05$ vs scrambled transfected cells treated with TWEAK. Results represent three independent experiments. Error bars represent the S.E.M.

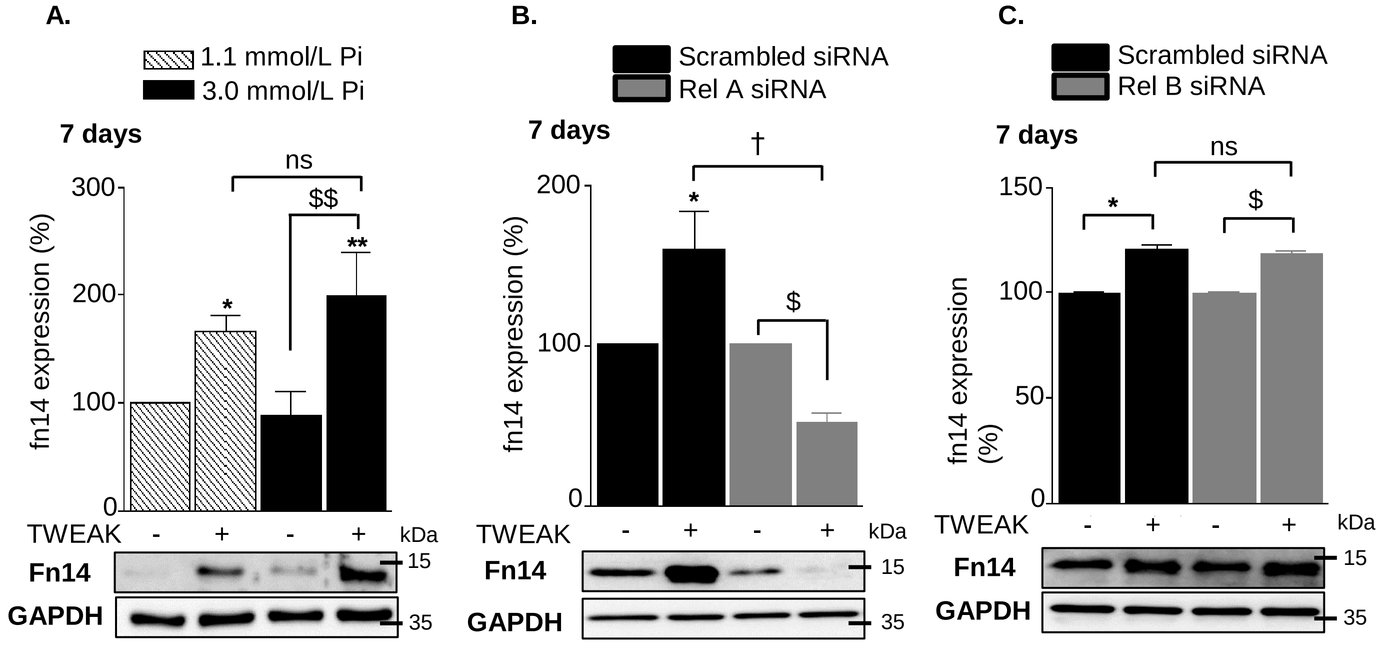

Supplemental figure 12. TWEAK promotes Fn14 expression through canonical and non-canonical activation of NFκB signaling in h-VSMCs. **A.** Immunoblotting of Fn14 and GAPDH performed on cell lysates prepared from h-VSMCs exposed for 7 days to 100ng/mL of TWEAK in presence of 1.1 or 3.0 mmol/L of Pi. * $p<0.05$ and ** $p<0.01$ vs. cells exposed to 1.1 mmol/L Pi without TWEAK. $$ $p<0.01$ vs. cells exposed to 3.0 mmol/L Pi without TWEAK. Results represent four independent experiments. Error bars represent the S.E.M. **B. and C.** Immunoblotting of Fn14 and GAPDH performed on cell lysates prepared from h-VSMCs transfected with either scrambled, Rel A (B.) or Rel B (C.) siRNA and exposed for 7 days to 100ng/mL of TWEAK in pro-calcific conditions (3.0 mmol/L Pi). * $p<0.05$ vs. scrambled transfected cells not exposed to TWEAK. $ $p<0.05$ vs. cells transfected with RelA or RelB siRNA and not exposed to TWEAK. † $p<0.05$ vs scrambled transfected cells treated with TWEAK. Results represent four independent experiments. Error bars represent the S.E.M.

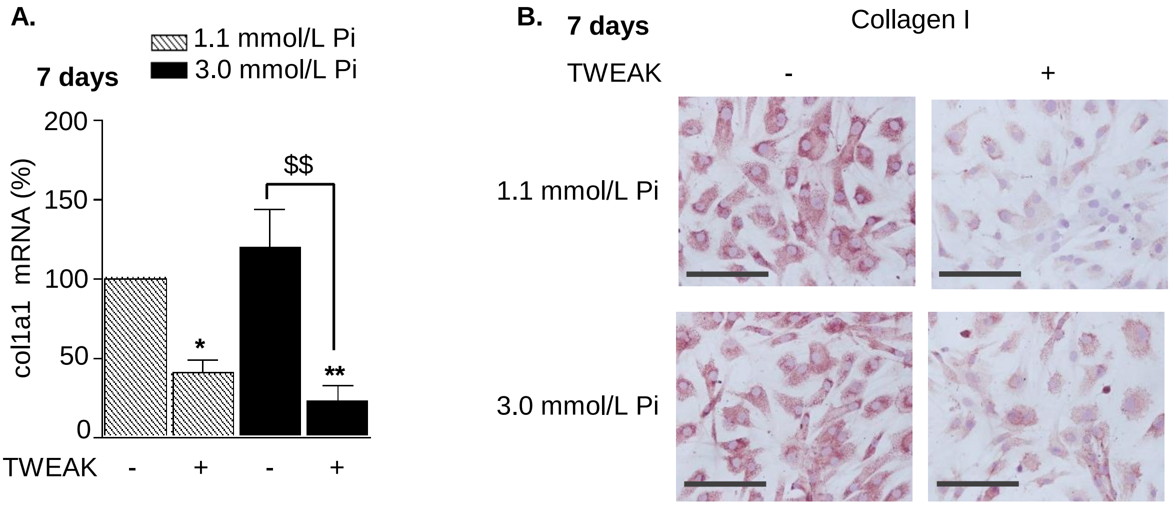

Supplemental figure 13. TWEAK reduces collagen expression in h-VSMCs during the mineralization process. The effects of TWEAK (100ng/mL, 7 days) were assessed both in non-calcific (Ct: 1.1 mmol/L Pi) and pro-calcific (Pi: 3.0 mmol/L Pi) conditions. **A.** Effects of TWEAK on col1a1 mRNA expression, assessed by qRT-PCR. * $p<0.05$, ** $p<0.01$ vs. cells exposed to 1.1 mmol/L Pi without TWEAK. $$ $p<0.01$ vs. cells exposed to 3.0 mmol/L Pi without TWEAK. Results represent three independent experiments performed in triplicate. Error bars represent the S.E.M. **B.** Effects of TWEAK on type I collagen protein expression, assessed by immunocytochemistry. Experiments were performed three times in triplicate. Images scale bars: 100µm.

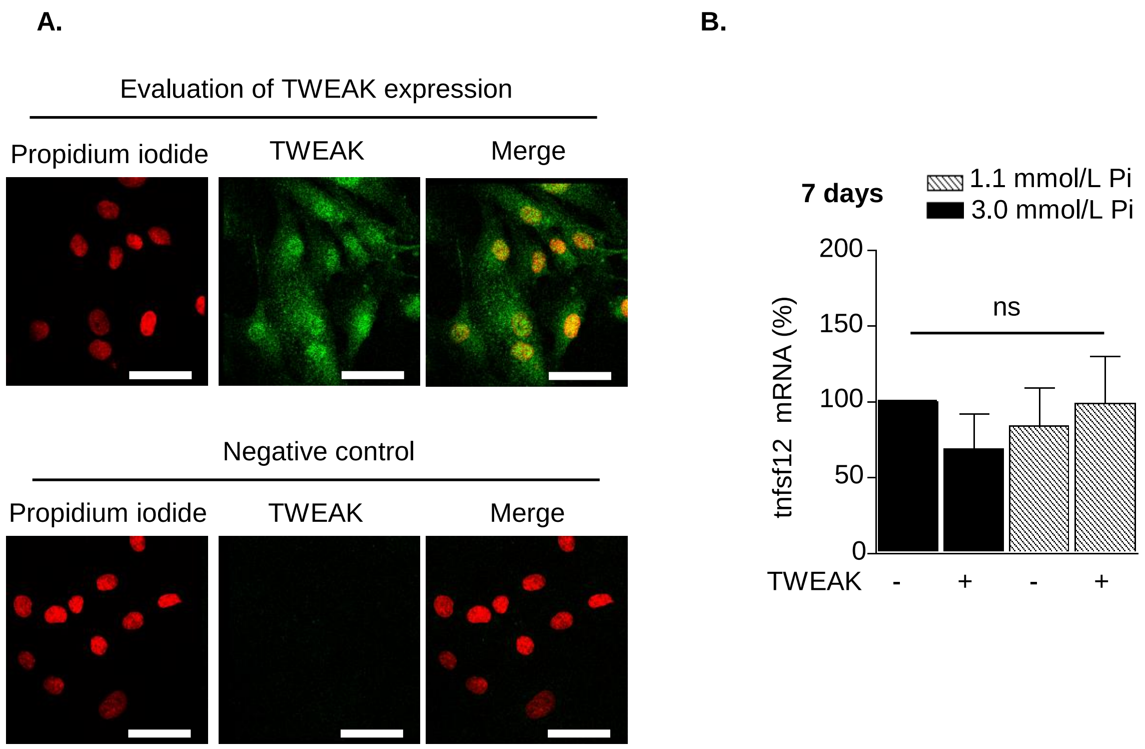

Supplemental figure 14. TWEAK expression is not modulated during the mineralization process. **A.** Evaluation of TWEAK protein expression in primary h-VSMCs assessed by immunocytochemistry. Negative control of immunostaining was performed without incubation of the primary antibody. Images scale bars: 50µm**. B.** Evaluation of TWEAK mRNA expression during the mineralization process evaluated by qRT-PCR. H-VSMCs were cultured for 7 days in non-calcific (1.1 mmol/L Pi) or pro-calcific conditions (3.0 mmol/L Pi) in presence or absence of 100 ng/mL TWEAK in medium containing 1% FBS. Results represent four independent experiments performed in triplicate. Error bars represent the S.E.M.

Methods from Supplemental Figures

Assessment of Mitosis and Apoptosis with propidium iodide

H-VSMCs (30 000 cells/well in 12 well plates) were treated in normal or procalcifying conditions for 3 days in presence or not of 100 ng/mL TWEAK. For assessment of mitosis and apoptosis, adherent cells were pooled with spontaneously detached cells, and stained in 100 µg/ml propidium iodide, 0.05% NP-40, 10 µg/ml RNAse A in PBS and incubated at 4°C for 3 h. This assay permeabilizes the cells and propidium iodide stains DNA. The percentage of apoptotic cells with decreased DNA staining (hypodiploid cells) and proliferating cells with increased DNA content (S+M) was counted by flow cytometry using FACS Canto cytometer and FACS Diva Software (BD Biosciences).

Morphological assessment of apoptosis

H-VSMCs (30 000 cells/well in 12 well plates) were treated in normal or procalcifying conditions for 3 days in presence or not of 100 ng/mL TWEAK. h-VSMCs monolayer on coverslips were then fixed in 4% paraformaldehyde for 10 min and PFA was quenched in 100 mmol/L glycin/PBS for additional 10 min. Nuclei were stained with DAPI (Sigma) to observe the typical morphological changes, as previously described.[1]

Assessment of early apoptosis and cell death

PE Annexin V apoptosis detection kit (BD Biosciences, San Diego) was used to evaluate whether h-VSMCs exposed to TWEAK and/or Pi experience early stages of apoptosis or cell death phenomena independent from apoptosis. After adequate treatment, h-VSMCs (30 000 cells/well in 12 well plates) were trypsinized and stained with PE-Annexin V and 7-AAD according to manufacturer’s instruction. This technic allow to quantify by flow cytometry the number of viable cells (Annexin V and 7-AAD negative), of cells that are in early apoptosis (Annexin V positive and 7-AAD negative) and of cells that are in late apoptosis or are already dead (positive for both Annexin V and 7-AAD). Cells

were analyzed using FACS Canto cytometer and FACS Diva Software (BD Biosciences). Staurosporine was used as a positive control for cell death.

Assessment of caspase 3 activity

After adequate treatment, caspase-3 activity (MBL, Nagoya, Japan) was measured following the manufacturer's instructions, as described previously.[2] In brief, cell extracts (70 mg proteins) were incubated with 200 mM DEVD-pNA or Ac-IETD-pNA and pNA light emission was quantified. Staurosporine was used as a positive control for cell death. Results are expressed as a percentage in comparison with the control condition (without treatment).

Assessment of cell survival (MTT)

MTT [3-(4,5-dimethylthiazolyl-2-yl)-2,5-diphenyltetrazolium bromide] (Sigma- Aldrich; St. Louis, MO) assay was used for evaluation the viability of h-VSMCs in response to the various treatment performed all over the study. Briefly, h-VSMCs (4000 cells/well in 96 well plates), transfected or not with either a scrambled siRNA or a siRNA targeting Fn14, RelA or RelB were cultured for 7 days in non-calcifying (1.1 mmol/L Pi) or pro-calcifying conditions (3 mmol/L Pi) in presence or absence of 100 ng/mL TWEAK and/or the MAPK inhibitor U0126. After stimulation, MTT solution was added to each well at a final concentration of 500 μg/ml for 2 h. Formazan crystals formed by living cells were then dissolved in DMSO and the absorbance was measured at 570 nm.

Immunostaining of type I collagen

After adequate treatment, h-VSMCs monolayer on coverslips was washed with PBS, fixed with 2% ice-cold paraformaldehyde (PFA) for 5 min at room temperature (RT), and permeabilized for 10 min at RT with 0.1% triton X-100 in PBS containing 1% BSA. Endogenous peroxidase activity and non-specific binding of the antibody were blocked by 0.3% H_2O_2 in PBS 1% BSA for 10 min at RT, and by incubation in a blocking solution (1% BSA in PBS) for 30 min at RT respectively. H-VSMCs monolayer was then incubated for 1 hr at RT with primary antibody (rabbit polyclonal IgG anti-

human-type I collagen, Thermo scientific PA1-26204, 1:200 dilution from original unit) in PBS containing 1% BSA. H-VSMCs were then rinsed and incubated with secondary antibody (anti-rabbit IgG (whole molecule)-peroxidase produced in goat, Sigma A6154, 1:500 dilution from original unit), prepared in PBS 1% BSA, for 1 hr at RT. Coverslips were then widely washed in PBS. For peroxidase detection, h-VSMCs were incubated with substrate kit for peroxidase (Vector® *NovaRED™* SK-4800) at RT until suitable staining develops. Coverslips were counterstained with hematoxylin solution and mounted on glass microscope slides using VectaMount™ mounting medium (Vector® H-5000).

Supplemental References

1. Ortiz A, Justo P, Sanz A, Melero R, Caramelo C, Guerrero MF, et al. Tubular cell apoptosis and cidofovir-induced acute renal failure. *Antivir Ther*. 2005;10:185-190
2. Justo P, Sanz AB, Sanchez-Niño MD, Winkles JA, Lorz C, Egido J, et al. Cytokine cooperation in renal tubular cell injury: The role of tweak. *Kidney Int*. 2006;70:1750-1758
